# Factors associated with severe COVID-19 in immunocompromised subgroups in England from 2020 to 2024: an OpenSAFELY cohort study

**DOI:** 10.1016/j.ebiom.2026.106327

**Published:** 2026-06-26

**Authors:** Edward P.K. Parker, Thomas Hartney, Linda Nab, Gayatri Amirthalingam, Nick Andrews, Eleanor V.H. Barry, Ian J. Douglas, Kathryn E. Mansfield, Anne Suffel, Meredith Leston, OpenSAFELY Collaborative, Brian MacKenna, William J. Hulme, Laurie A. Tomlinson

**Affiliations:** a London School of Hygiene and Tropical Medicine, Keppel Street, London, WC1E 7HT, UK; b NIHR Health Protection Research Unit in Vaccines and Immunisation, London School of Hygiene and Tropical Medicine, London, UK; c Department of Datascience & Biostatistics, Julius Center for Health Sciences and Primary Care, University Medical Center Utrecht, Utrecht, the Netherlands; d UK Health Security Agency, London, UK; e School of Health and Care Sciences, University of Lincoln, Brayford Pool, Lincoln, LN6 7TS, UK; f Nuffield Department of Primary Care Health Sciences, University of Oxford, Oxford, OX2 6CG, UK; g Bennett Institute for Applied Data Science, Nuffield Department of Primary Care Health Sciences, University of Oxford, OX2 6GG, UK

**Keywords:** COVID-19, Burden, Immunocompromise, Immunosuppression, Vaccine

## Abstract

**Background:**

Individuals with compromised immune systems are particularly vulnerable to COVID-19. Although several studies have assessed factors associated with COVID-19-related hospitalisation and mortality in the general population, few studies have focused specifically on immunocompromised populations.

**Methods:**

With the approval of NHS England, we conducted a cohort study using the OpenSAFELY platform covering successive waves of COVID-19 (wave 1, alpha, delta, omicron BA.1/BA.2, JN.1). In each wave, we included adults in five hierarchically assigned immunocompromised subgroups: solid organ transplant (SOT); bone marrow compromise (BMC); active radio- or chemo-therapy (RCT); active immunosuppressive medication (IMM); and primary or acquired immunodeficiency (IMD). In each subgroup, we estimated wave-specific rates of severe COVID-19-related outcomes and relative hazards according to vaccination status, sociodemography (age, ethnicity, deprivation), and comorbidities, as determined at the start of the wave.

**Findings:**

We included between 475,360 (BA.1/BA.2 wave) and 508,545 (JN.1 wave) immunocompromised individuals per wave. Across successive waves, individuals with SOT, BMC, or RCT exhibited higher rates of severe COVID-19 than those with IMM or IMD. Compared with being unvaccinated in the past 26 weeks, vaccination in the 12 weeks preceding the start of a wave was associated with a consistent reduction in severe COVID-19, albeit with a smaller effect size among SOT recipients relative to other subgroups. Though attenuated compared to earlier waves, this association persisted during JN.1 dominance (adjusted hazard ratios of 0.88 [95% CI 0.60–1.30] for SOT, 0.64 [95% CI 0.53–0.77] for BMC, 0.68 [95% CI 0.44–1.03] for RCT, 0.68 [95% CI 0.52–0.88] for IMM, and 0.68 [95% CI 0.55–0.82] for IMD). Older age and increased comorbidity count were strongly associated with increased risk of severe COVID-19 across subgroups and waves.

**Interpretation:**

The risk of COVID-19 varies substantially within and across immunocompromised subgroups. The presence of other comorbidities was strongly associated with the risk of severe COVID-19 across successive waves. Primary vaccination and successive booster doses were associated with a consistent reduction in severe COVID-19 in this high-risk population, including during the JN.1 wave.

**Funding:**

National Institute for Health and Care Research (NIHR) Health Protection Research Unit in Vaccines and Immunisation (NIHR200929/NIHR207408).

**Research in context**

**Evidence before this study**

We searched MEDLINE for studies investigating factors associated with COVID-19 risk in immunocompromised populations in the UK between 1st December 2020 and 1st October 2025 using the following term: “(coronavir∗ or covid∗ or sars∗) and (immunosuppress∗ or immunocomp∗ or immunodeficien∗) and risk and (UK or United Kingdom or England or Northern Ireland or Scotland or Wales)”. Several studies reported on COVID-19 vaccine effectiveness, either aggregated across immunocompromised populations or focusing on a specific subgroup. We identified multiple studies quantifying absolute or relative risk of severe COVID-19 in different immunocompromised subgroups. A higher risk has consistently been observed among transplant recipients and individuals with haematological cancer compared with other immunocompromised subgroups. Several studies have also explored heterogeneity in risk within a specific immunocompromised subgroup, although these have generally focused on clinical features such as the type and timing of diagnoses and treatments. Few studies have looked at a broader range of clinical and sociodemographic factors when assessing variation in risk within and across immunocompromised populations.

**Added value of this study**

This study assessed variation in the risk of severe COVID-19 outcomes in immunocompromised adults in England across successive waves of COVID-19. In each wave, individuals were assigned to one of five immunocompromised subgroups (solid organ transplant, bone marrow compromise, radio- or chemo-therapy, active immunosuppressive medication, primary or acquired immunodeficiency). In each subgroup and in each wave, we assessed the absolute rate and relative hazard of severe COVID-19 outcomes according to sociodemographic factors (age, ethnicity, deprivation), comorbidities (diabetes mellitus, chronic kidney disease, etc), and vaccination status. We found older age and comorbidity count to be associated with an increased risk of severe COVID-19 that persisted across waves. Compared with being unvaccinated in the past 26 weeks, vaccination in the 12 weeks preceding the start of a wave was associated with a consistent reduction in the risk of severe COVID-19. During the JN.1 wave from December 2023, recent vaccination was associated with a 12% reduction in severe COVID-19 outcomes among solid organ transplant recipients and a 32–36% reduction in other subgroups.

**Implications of all the available evidence**

The risk of COVID-19 varies within and across different subgroups of immunocompromised adults. The results of our study highlight that individuals with solid organ transplants, bone marrow compromise, and recent treatment with radio- or chemo-therapy continue to be at greater risk than other immunocompromised subgroups. The presence of clinical comorbidities such as diabetes and chronic kidney disease (among others) have been strongly associated with the risk of severe COVID-19 outcomes across successive waves. Booster vaccine doses have consistently been associated with a reduction in severe COVID-19 outcomes, including those administered in the Autumn 2023 campaign.

## Introduction

People with compromised immune systems have endured a disproportionate burden of morbidity and mortality from COVID-19. In the UK, immunocompromised individuals make up approximately 4% of the population but accounted for 22% of COVID-19-related deaths in 2023.^1^ Key measures to mitigate this burden have included shielding to reduce infection exposure, repeated vaccination, and eligibility for therapeutic antibodies and antivirals.^2^

Immunocompromised individuals comprise a heterogeneous group. A recent meta-analysis showed that, compared with immunocompetent individuals, excess risk of COVID-19-related mortality was highest among transplant recipients and individuals with haematological cancer (pooled odds ratios relative to immunocompetent individuals of 2.12 [95% CI 1.50–2.99] and 2.02 [95% CI 1.69–2.42], respectively), but only marginally greater in people with rheumatic disease (pooled odds ratio 1.28 [95% CI 1.13–1.45]).^3^ Marked variation in risk has also been seen in observational cohort studies stratifying individuals by the type and timing of their immunosuppressive conditions and treatments.^1^^,^^4^ For example, adjusted incidence rate ratios for COVID-19-related hospitalisations in 2023 were notably higher for individuals with haematologic malignancy undergoing active treatment (9.69 [95% CI 8.44–11.12]) compared to those with no treatment in the previous 12 months (2.95 [95% CI 2.39–3.63]).^1^ However, studies of broader sociodemographic and clinical factors associated with COVID-19 across immunocompromised subgroups are warranted.

COVID-19 vaccination in the UK commenced in December 2020.^2^^,^^5^ Immunocompromised individuals became eligible for a third primary dose in September 2021, and have since been offered additional doses via annual Spring and Autumn booster campaigns. The immunogenicity^6^^,^^7^ and effectiveness^8^^,^^9^ of COVID-19 vaccines are attenuated in immunocompromised compared with immunocompetent individuals. Booster doses increase the likelihood of seroconversion^7^^,^^10^ and reduce the risk of COVID-19-related hospitalisation among immunocompromised individuals.^8^^,^^11^ However, the extent to which vaccine effectiveness varies across immunocompromised subgroups, over successive booster campaigns, and in response to emerging variants is unclear.

We aimed to determine factors associated with severe COVID-19 in different subgroups of immunocompromised individuals in England between 2020 and 2024.

## Methods

### Data sources

All data were linked, stored and analysed securely using the OpenSAFELY platform, https://www.opensafely.org/, as part of the NHS England OpenSAFELY COVID-19 service. Data include pseudonymised data such as coded diagnoses, medications and physiological parameters. No free text data are included. Detailed pseudonymised patient data are potentially re-identifiable and therefore not shared. The dataset analysed within OpenSAFELY-TPP is based on 24 million people registered with GP surgeries using SystmOne, a GP IT System supplied by the health technology company TPP. Primary care records managed by the GP software provider TPP were linked through OpenSAFELY to records of inpatient hospital admission via NHS Digital’s Hospital Episode Statistics (HES) and national death registry records from the Office for National Statistics (ONS). All code is shared openly for review and re-use under MIT open licence (https://github.com/opensafely/covid-risk-immunocompromised).

The OpenSAFELY platform was developed in response to the COVID-19 pandemic^12^ and has been extensively used for analyses of disease burden,^13^^,^^14^ healthcare utilisation,^15^ and vaccine effectiveness,^5^^,^^16^^,^^17^ among others. OpenSAFELY-TPP is broadly representative of the English population in terms of age, sex, deprivation index, ethnicity, and primary causes of death.^18^

### Ethical approval

This study was approved by the Health Research Authority (REC reference 20/LO/0651) and by the London School of Hygiene & Tropical Medicine’s Ethics Board (reference 21863).

### Study population

**Fig. 1:**
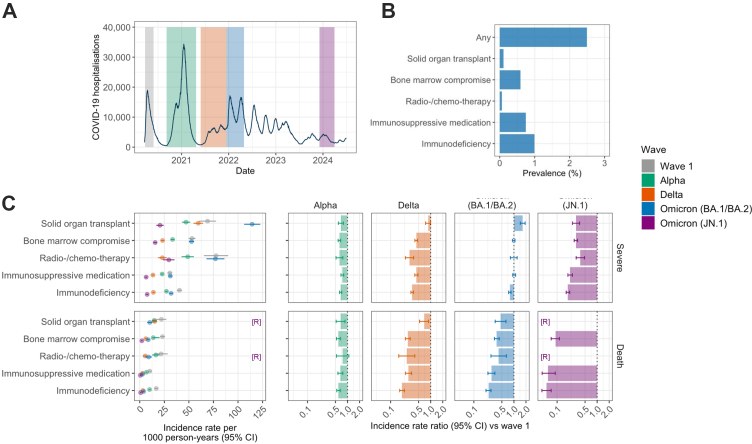
**Absolute risk of severe COVID-19 over time byimmunocompromisedsubgroup. (A)** Study timeline. Counts of confirmed COVID-19-related hospitalisations in England were obtained from Our World in Data (up to 08 December 2023) and the UK Health Security Agency (09 December 2023 onwards). Waves are highlighted by shaded areas. **(B)** Prevalence of assigned immunocompromised subgroups at the start of the Omicron wave (15 December 2021). Individuals were assigned to subgroups based on decreasing anticipated risk of COVID-19-related mortality as follows: solid organ transplant > bone marrow compromise > radio-/chemo-therapy > immunosuppressive medication > immunodeficiency. The denominator for these estimates was obtained from Nab et al.^13^**(C)** Absolute rates of severe COVID-19-related outcomes by immunocompromised subgroup over time. [R]; redacted due to small counts.

We defined five cohorts covering the following waves of COVID-19: wave 1 from 23 March to 30 May 2020; the alpha B.1.1.7 wave from 07 September 2020 to 24 April 2021; the delta B.1.617.2 wave from 28 May to 14 December 2021; the omicron BA.1/BA.2 wave from 15 December 2021 to 29 April 2022; and the JN.1 wave from 04 December 2023 to 31 March 2024 (Fig. 1A). Each wave was analysed separately and individuals could be included for more than one wave. The start and end dates of the first four waves reflect those used in our previous study of COVID-19-related mortality in the general population.^13^ The start date of the JN.1 wave reflects the first date at which this variant became nationally dominant in UK Health Security Agency (UKHSA) genomic surveillance records, while the end date follows the sustained reduction in COVID-19-related hospitalisations in March 2024.^19^

Criteria and codelists used to define variables are provided in Supplementary Table S1. Baseline sociodemographic and clinical characteristics were defined based on primary care records up to and including the first day of each wave (index date). Each cohort comprised individuals with at least one indicator of immunocompromise in their primary care records by the index date. Subgroups of interest included: (i) any history of solid organ transplant (SOT); (ii) any history of bone marrow compromise (BMC), including haematologic malignancy or bone marrow transplant; (iii) radiotherapy or chemotherapy (RCT) in the past 6 months (for both cancer and non-cancer usage); (iv) immunosuppressive medication (IMM) in the past 6 month; or (v) any history of primary or acquired immunodeficiency (IMD). We further subcategorised subgroups based on the type and timing of immunocompromise (Supplementary Table S1). Individuals were included if they: were aged 18–110 years; had been registered with a TPP practice for at least 3 months before the index date; had complete data on sex, NHS region, and index of multiple deprivation; did not have an outcome or censoring event on index date; and had no documented SARS-CoV-2 infection in the preceding 90 days (to ensure that observed outcomes did not have an onset date preceding the start of the wave).

### Outcomes

We considered two nested outcomes: severe COVID-19 (a composite of COVID-19-related hospitalisation, COVID-19-related attendance at an accident and emergency department, and COVID-related mortality); and COVID-19-related mortality (Supplementary Table S1).

### Covariates

We categorised participants into sociodemographic and clinical subgroups that aligned with our prior analysis of COVID-19-related mortality in the general population.^13^ Sociodemographic factors included age, sex, ethnicity, region, deprivation quintile, care home residence, and smoking status. Clinical factors included underlying health conditions associated with increased risk of COVID-19.^2^ We further defined comorbidity count based on: chronic respiratory disease (including asthma); chronic cardiac disease; chronic kidney disease; chronic liver disease; chronic neurological disease or learning disability; diabetes mellitus; asplenia; severe obesity; and severe mental illness.

We categorised COVID-19 vaccination history based on number of doses and time since last dose (0–12 weeks, 13–26 weeks, and 27+ weeks or unvaccinated). We did not differentiate between COVID-19 vaccine products. We categorised prior infection status based on the most recent wave of infection (Supplementary Table S1).

We coded people with missing ethnicity data as unknown; those with missing BMI records as having no obesity; and those with missing smoking information as non-smokers (assuming that obesity and smoking would be more likely to be recorded if present). For other variables, the absence of codes was interpreted as absence of the condition or event.

### Statistical analysis

We performed an initial descriptive analysis of the intersection of immunocompromised subgroups as recorded in primary care. All other analyses were stratified by subgroup. To ensure subgroups were mutually exclusive, we assigned individuals hierarchically according to anticipated decreasing risk as follows: SOT (highest risk); BMC; RCT; IMM; and IMD (lowest risk). This risk hierarchy was informed by prior reviews^3^^,^^20^ alongside our previous studies of COVID-19-related mortality in the general population.^13^^,^^14^ For each wave, we calculated crude outcome rates per 1000 person-years overall and stratified by sociodemographic and clinical subgroups. 95% confidence intervals (CIs) were calculated based on a normal approximation of the binomial distribution.

We used Cox proportional hazards models to estimate hazard ratios (HRs) and 95% CIs for the association between each covariate and each COVID-19-related outcome, with time since the start of the wave as the underlying timescale. For each covariate, we fitted: (i) minimally adjusted models adjusting for age (using a restricted cubic spline with four knots) and sex; (ii) extended models further adjusting for time since last vaccine dose and prior infection; and (iii) fully adjusted models further adjusting for ethnicity, deprivation quintile, and comorbidity count. All models included region as a stratification variable to account for geographic variation in infection rates. Follow-up continued until the earliest of death, deregistration, the end of the wave, or the outcome of interest. HRs should be interpreted as a weighted average of the time-varying HRs in each wave-specific follow-up period.^21^ Given that each wave was defined and analysed separately, associations should be interpreted as specific to the population included in a given wave. We performed a sensitivity analysis censoring on receipt of a COVID-19 vaccine dose during follow-up. As a precaution against small number disclosures, we rounded counts to the nearest 5 and redacted rounded counts of ≤10, alongside any derived metrics. Additional redactions were applied to ensure that redacted values could not be derived. Our findings are reported in accordance with RECORD guidelines.^22^

### Patient and public involvement

Our interpretations are informed by a public involvement workshop with immunocompromised participants in 2024.^23^

### Role of funders

The funders had no role in the study design, data collection, data analysis, data interpretation, writing of the report, or the decision to submit for publication.

## Results

### Study population

We identified 481,990 immunocompromised individuals in wave 1, 479,485 in the alpha wave, 484,565 in the delta wave, 475,360 in the BA.1/BA.2 wave, and 508,545 in the JN.1 wave (Supplementary Table S2). This corresponds to an overall prevalence of immunocompromised individuals of ∼2.5% based on denominators from our prior analyses^13^ (Fig. 1B).

Prior to hierarchical assignment of immunocompromised subgroups, we observed common intersections in primary care coding between BMC and IMD (reflecting the presence of haematological malignancies in both underlying codelists), IMD and IMM (reflecting multiple treatment/diagnosis combinations including immunosuppressive therapies for autoimmune/inflammatory rheumatic disease), and SOT and IMM (Supplementary Fig. S1 and Table S3). Notably, 58% of individuals with SOT had no other primary codes indicative of immunocompromise (IMM or other).

**Fig. 2:**
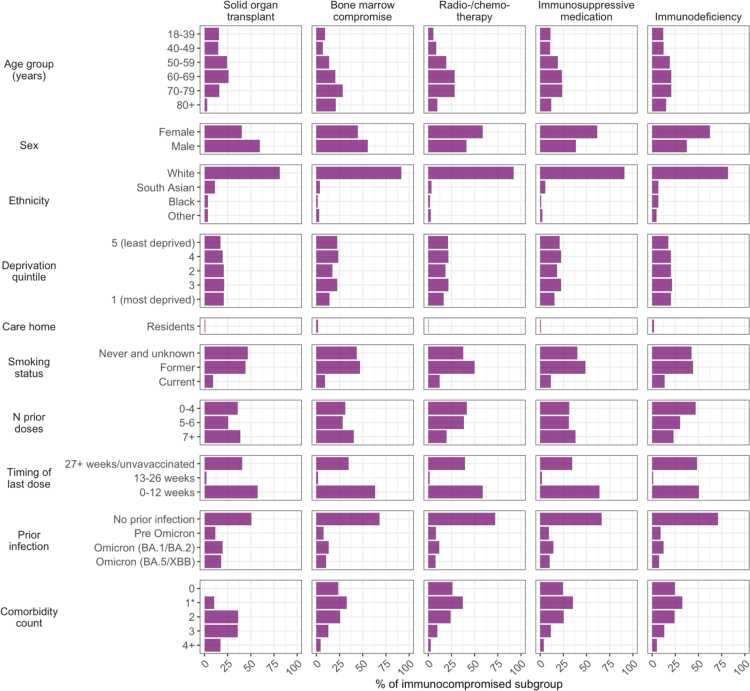
**Baseline characteristics byimmunocompromisedsubgroup.** Data shown for the start of the JN.1 wave (04 December 2023). See Supplementary File S1 for underlying date.

Baseline sociodemographic and clinical characteristics were consistent across waves (Supplementary Table S4 and File S1). For example, in the JN.1 wave, after hierarchical assignment of subgroups, we identified 21,350 (4.2%) individuals with SOT, 120,300 (23.7%) with BMC, 12,615 (2.5%) with RCT, 148,815 (29.3%) with IMM, and 205,465 (40.4%) with IMD. Individuals with SOT were younger and had more comorbidities compared with other subgroups (Fig. 2). Individuals with BMC were older than other subgroups. Care home residence ranged from 0.3% in individuals with RCT to 2% in individuals with IMD.

### Absolute incidence of COVID-19 outcomes

In wave 1, the crude incidence rates of severe COVID-19 per 1000 person-years were 69.0 (95% CI 60.7–77.3) for SOT, 53.2 (50.1–56.4) for BMC, 77.7 (65.6–89.7) for RCT, 30.6 (28.5–32.6) for IMD, and 40.4 (38.2–42.5) for IMM (Fig. 1C; Supplementary Table S5; see Supplementary Fig. S3 for age-stratified estimates). In the alpha wave, there was a modest decline in severe COVID-19 across subgroups (incidence rate ratio [IRR] relative to wave 1 of 0.6–0.7). A further decline was apparent in the delta wave, which was preceded by widespread primary vaccination (IRRs relative to wave 1 of 0.3–0.4), with the exception of individuals with SOT (IRR of 0.9). The incidence of severe COVID-19 rose again in the BA.1/BA.2 wave, with a larger rise in individuals with SOT than other subgroups. In the JN.1 wave, the incidence of severe COVID-19 per 1000 person-years ranged from 6.5 (5.8–7.2) for IMM to 29.6 (24.2–35.0) for RCT (IRRs relative to wave 1 of 0.2–0.4 across subgroups).

The crude incidence rates of COVID-19-related mortality per 1000 person-years ranged from 10.2 (9.0–11.4) for IMM to 23.1 (21.1–25.2) for BMC in wave 1. There was a decline in COVID-19-related mortality rates relative to wave 1 across all subgroups in successive waves, albeit attenuated in the omicron BA.1/BA.2 wave (Fig. 1C).

### Association between recent vaccination and COVID-19

**Fig. 3:**
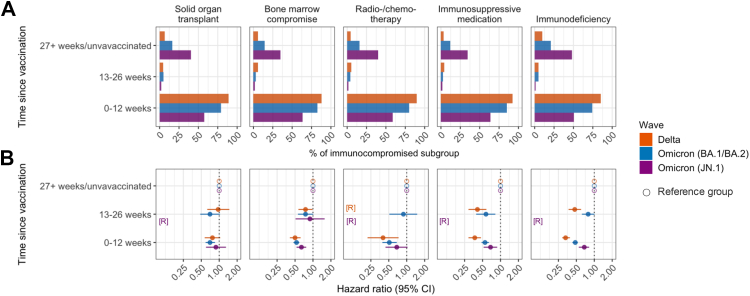
**Association between recent vaccination and severe COVID-19. (A)** Baseline vaccination status at the start of each wave after COVID-19 vaccine implementation. **(B)** Relative hazard of severe COVID-19 based on models adjusting for age, sex, prior infection, deprivation quintile, and comorbidity count, with region as a stratification factor. CI, confidence interval; [R] redacted due to small counts.

At the start of the delta wave (28 May 2021), 85% of individuals in our study population had received 2 doses of COVID-19 vaccine, 8% had received 1 dose, and 7% were unvaccinated. Most individuals had received their most recent dose in the preceding 12 weeks (88% overall; Fig. 3A). Compared with unvaccinated individuals, vaccination in the 12 weeks preceding the start of the delta wave was associated with a reduction in severe COVID-19, with fully adjusted HRs (95% CI) of 0.77 (0.57–1.04) for SOT, 0.50 (0.41–0.62) for BMC, 0.40 (0.22–0.73) for RCT, 0.37 (0.29–0.47) for IMM, and 0.33 (0.29–0.39) for IMD (Fig. 3B). Incidence rates and HRs for all waves and outcomes are provided in Supplementary File S2.

At the start of the omicron BA.1/BA.2 wave (15 December 2021), 80% of individuals had received their most recent (predominantly third) dose in the past 12 weeks (ranging from 74% in IMD to 85% in IMM). Compared with individuals who were unvaccinated in the past 26 weeks, recent vaccination (0–12 weeks) was associated with a 50% reduction in severe COVID-19 across individuals with BMC, RCT, IMM, and IMD (HRs of 0.48–0.55 based on fully adjusted models; Supplementary Table S6), with a significant but more modest reduction among individuals with SOT (HR 0.70 [0.59–0.85]).

At the start of the JN.1 wave (04 December 2023), 32% of our study population had received ≥7 doses (Fig. 2). Overall, 58% of individuals had received their most recent dose in the past 12 weeks, reflecting uptake of the Autumn 2023 booster campaign (ranging from 50% for IMD to 64% for IMM and BMC). Compared with individuals who were unvaccinated in the past 26 weeks, recent vaccination (0–12 weeks) was associated with a 32–36% reduction in severe COVID-19 across individuals with BMC, RCT, IMM, and IMD (HRs of 0.64–0.68 based on fully adjusted models; Supplementary Table S6). A corresponding HR of 0.88 (0.60–1.30) for recent vaccination was observed for individuals with SOT.

HRs associated with recent vaccination were consistent across sequentially adjusted models, and in sensitivity analyses censoring on receipt of a subsequent dose (Supplementary Table S6). Higher dose counts were associated with a reduction in the hazard of severe COVID-19 (Supplementary Fig. S4).

Outcome counts were generally insufficient to estimate associations between vaccination and COVID-19-related mortality. Where counts were sufficient (for common subgroups during the delta and BA.1/BA.2 waves), recent vaccination was associated with a larger reduction in COVID-19-related mortality than observed for severe COVID-19 (Supplementary Table S7).

### Prior infection

Prior infections in primary or secondary care settings had been recorded in 1% of individuals at the start of the alpha wave, 5% at the start of the delta wave, 8% at the start of the BA.1/BA.2 wave, and 32% at the start of the JN.1 waves (Fig. 2) – reflecting marked underestimation of true infection rates.^24^ Associations between prior infection and severe COVID-19 varied by subgroup and wave (Supplementary Fig. S5). Prior infection was associated with a reduction in severe COVID-19 only during the delta and BA.1/BA.2 waves, coinciding with a period of improved ascertainment due to free universal testing (between April 2021 and March 2022).

### Sociodemographic factors

**Fig. 4:**
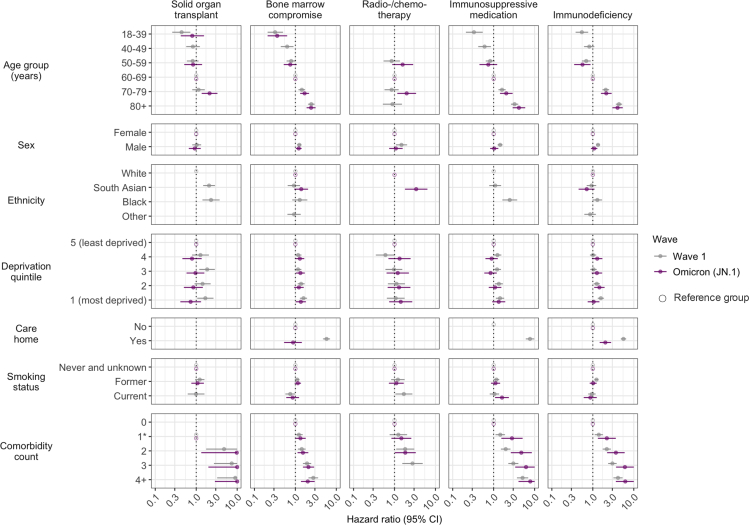
**Clinical andsociodemographicfactorsassociated withsevere COVID-19 in wave 1 and the JN.1 wave.** Relative hazard of severe COVID-19 based on models adjusting for age, sex, prior infection, deprivation quintile, and comorbidity count, with region as a stratification factor. Unplotted values indicate that estimates were redacted due to small counts or for secondary disclosure mitigation. CI, confidence interval.

In wave 1, age was strongly associated with the risk of severe COVID-19 across individuals with BMC, IMM, and IMD (e.g., fully adjusted HR of 3.24 [2.64–3.98] among individuals with IMM aged ≥80 years compared with those aged 60–69 years; Fig. 4). This gradient was not apparent among individuals with SOT and RCT in wave 1. However, by the JN.1 wave, older age was strongly associated with severe COVID-19 in all subgroups (Supplementary Fig. S6).

Disparities in severe COVID-19 by ethnicity varied across subgroups. In wave 1, among individuals with SOT, a higher hazard of severe COVID-19 was apparent among South Asian and Black compared with White individuals (fully adjusted HRs: South Asian, 2.06 [1.48–2.87]; Black, 2.30 [1.42–3.73]; Fig. 4). A similar disparity was evident for IMM among Black compared to White individuals (HR 2.47 [1.63–3.75]). By contrast, there was no evidence of disparities by ethnicity in individuals with BMC or IMD. In later waves, we observed an attenuation of disparities where evident in wave 1 (Supplementary Fig. S7). In some instances, inverse trends were evident (e.g., in the BA.1/BA.2 wave among individuals with IMD), which has also been seen in the general population.^13^

In wave 1, greater deprivation was associated with a higher hazard of severe COVID-19 (Fig. 4), albeit not among individuals with RCT. By the delta wave, deprivation differences were no longer evident among individuals with SOT (Supplementary Fig. S8). For individuals with BMC, IMD, and IMM, trends persisted into the delta, BA.1/BA.2, and JN.1 waves, albeit with wide CIs in the latter due to low outcome counts.

Care home residence was strongly associated with severe COVID-19 in wave 1 (fully adjusted HRs of 5.77 [4.74–7.02] for BMC, 7.71 [6.08–9.78] for IMM, and 5.60 [4.83–6.51] for IMD; Fig. 4). By the JN.1 wave, this association was no longer evident for individuals with BMC (HR 0.88 [0.53–1.44]) and persisted with attenuated effect size for individuals with IMD (HR 2.02 [1.47–2.79]; Fig. 4). Outcome counts were insufficient to estimate HRs for individuals with SOT or RCT.

### Comorbidities

Across all waves and subgroups, there was an incremental increase in the hazard of severe COVID-19 with increasing comorbidity count. In wave 1, compared with individuals with no recorded comorbidities, individuals with 3 comorbidities had a 2–7-fold higher hazard of severe COVID-19 (Fig. 3). These differences persisted in later waves (Fig. 4 and Supplementary Fig. S9). Numerous underlying health conditions (e.g., chronic respiratory disease, chronic kidney disease) appear to have contributed to this incremental risk, with adjusted HRs in the range of 1.5–3, albeit with variation across subgroups and waves (Supplementary Fig. S10).

### Type and timing of immunocompromise

Among individuals with SOT, BMC, or IMD, we observed a higher hazard of severe COVID-19 if the most recent record of immunocompromise-related diagnosis or treatment fell within the past 12 months versus more than 12 months ago (Supplementary Fig. S11A). Among SOT recipients, the hazard of severe COVID-19 was lower in individuals with non-kidney versus kidney transplants (fully adjusted HR 0.57 [0.41–0.80] in wave 1; Supplementary Fig. S11B). Among individuals with BMC, those without bone marrow transplants had a lower hazard of severe COVID-19 compared with transplant recipients in the JN.1 wave (HR 0.57 [0.41–0.79]) but not in earlier waves (Supplementary Fig. S11C).

### COVID-19-related mortality

Incidence rates and HRs for analyses of COVID-19-related mortality are provided in Supplementary File S2. Where outcome counts were sufficient, associations reflected those observed for severe COVID-19, although there was a general tendency (e.g., for age and vaccination status) for stronger effect sizes with COVID-19-related mortality as an endpoint.

## Discussion

Immunocompromised individuals are routinely considered as a single group in public health policy and research.^2^^,^^8^^,^^25^ We distinguished between five immunocompromised subgroups, of which three (SOT, BMC, and RCT) exhibited a higher absolute risk of severe COVID-19 compared with the remaining two (IMM and IMD). Across successive waves of COVID-19, older age, care home residence, and higher comorbidity count were strongly associated with severe COVID-19. Recent vaccination was associated with a reduction in severe COVID-19, including during the Autumn 2023 booster campaign preceding emergence of the JN.1 variant.

The five subgroups in our study align with those established in a recent scoping review,^20^ although we applied a different order of anticipated risk (SOT > BMC > RCT > IMM > IMD as opposed to BMC > SOT > IMM > RCT > IMD) informed by our prior studies in OpenSAFELY.^13^^,^^14^ The observed disparities in COVID-19 outcomes were broadly consistent with our anticipated hierarchy, and may reflect a greater degree of immunosuppression in higher-risk subgroups in contrast to a more heterogeneous immune depletion in other subgroups.

The INFORM study compared rates of severe COVID-19 between immunocompromised and non-immunocompromised individuals in England during 2022^4^ and 2023.^1^ Findings were consistent with the risk hierarchy across immunocompromised subgroups reported here. In addition, among transplant recipients in the 2023 INFORM cohort, the risk of severe COVID-19 was higher if transplants had been received in the past year compared with >1 year ago,^1^ consistent with the present study.

Recent vaccination was associated with a reduction in severe COVID-19 across immunocompromised subgroups. Our study was not designed as a causal analysis of vaccine effectiveness, but as a series of cohorts in which baseline characteristics were defined at the start of each wave. The timing of later waves was such that each started ∼3 months after the launch of a distinct vaccination campaign. Our models included covariates typically adjusted for as confounders in observational studies of vaccine effectiveness.^8^^,^^16^^,^^26^ While acknowledging the potential for residual confounding (e.g., healthy vaccinee bias resulting in a shift in HRs for recent vaccination away from the null) and the fact that the start of follow-up was not aligned with vaccination, our findings are consistent with an incremental beneficial population-level effect of successive booster doses in immunocompromised individuals. There was a modest attenuation in the protective association over time. A similar finding of attenuated relative effectiveness against following the Autumn 2023 booster campaign was reported in a test-negative case control study of the general population, with effectiveness peaking at 50.6% (95% CI 44.2–56.3%) 2–4 weeks after boosting.^27^ Attenuated effectiveness during the JN.1 wave may reflect the accumulation of baseline immunity following repeated infection and vaccination, as well as enhanced immune escape of the JN.1 variant compared to prior lineages.^28^

The incremental benefits of recent vaccination were smaller among SOT recipients compared with other subgroups. All HRs were consistent with a protective effect, but this was only statistically significant for the BA.1/BA.2 wave. Prior observational studies of COVID-19 vaccine immunogenicity have documented lower seroconversion rates and post-vaccination antibody levels in SOT recipients compared to other immunocompromised subgroups,^6^^,^^29^ which may contribute to attenuated protection against clinical endpoints.

Comorbidity count was associated with a steep incremental increase in the risk of severe COVID-19. This effect was consistent across subgroups and waves. Although similar patterns have been documented in the general population,^13^^,^^14^^,^^30^^,^^31^ the additive effect in a population already known to be at heightened risk of COVID-19 is striking. Disparities in severe COVID-19 according to ethnicity and deprivation index varied over time and across immunocompromised subgroups, likely reflecting the complex interplay of factors including structural discrimination, inequitable care access, and variable exposure in occupational, household, and clinical settings.^13^^,^^24^^,^^25^ The heterogeneity in findings across subgroups highlights the importance of tailored efforts to understand the determinants of sociodemographic disparities in infection risk.

Strengths of our study include the scale and representativeness of OpenSAFELY,^18^ offering the power to explore severe outcomes in narrow population subgroups. Our stratified analysis approach ensured that associations were not driven by numerically dominant subgroups. By highlighting a subgroup-specific protective association for recent vaccination that persisted across booster campaigns, our findings have the capacity to enhance confidence in the vaccination programme. This is particularly pertinent in the context of concerns around vaccine fatigue^32^^,^^33^ and the diminishing returns in vaccine coverage observed across campaigns.

Our study has several limitations. We did not include data on monoclonal antibodies and antivirals, which were deployed in the UK from December 2021 to reduce the risk of disease progression after SARS-CoV-2 infection in high-risk groups. Immunocompromised subgroups were defined based on the presence of diagnostic and prescription codes in primary care. Although most prescriptions in England are supplied by general practitioners, some medicines, including targeted immunosuppressive medications (i.e., biologics), are largely supplied by hospitals.^34^ The OpenSAFELY platform previously provided access to a novel dataset of high-cost drugs supplied by hospitals during the pandemic but access to these important data has not continued outside of the immediate COVID-19 emergency.^35^^,^^36^ While our approach is likely to capture several groups with high sensitivity (e.g., transplant recipients^37^), subgroups defined by treatments given in secondary care (particularly RCT recipients) are likely to be under-represented in our study and may lack generalisability if individuals with primary care coding are not representative of the target populations. RCT recipients may also lack generalisability if treatment is not recorded or updated in primary care records in a timely fashion. Steroid recipients are considered immunocompromised depending on the dose and duration of treatment,^2^ but were not included given the challenges of accurately determining daily dosages from prescription records. Finally, we lacked virological testing data to confirm that severe outcomes were the result of acute episodes rather than longer-term manifestations of COVID-19 including potential progression of other health conditions.^38^

Overall, our findings highlight substantial variation in the risk of severe COVID-19 within and between immunocompromised individuals. We documented three subgroups (SOT, BMC, and RCT) exhibiting a higher absolute risk of severe COVID-19 compared with the remaining groups (IMM and IMD). Older age, time since diagnosis, and comorbidity count were strongly and persistently associated with severe COVID-19 across subgroups. Successive booster campaigns appear to offer an incremental protective effect that persisted in the Autumn 2023 booster campaign. Improving vaccine coverage in future campaigns, especially in those at greatest risk of severe disease, presents a crucial opportunity to curb the ongoing risk of severe COVID-19 in immunocompromised individuals.

## Contributors

Conceptualisation: EPKP, LAT; Methodology: EPKP, TH, LN, WJH, LAT, ML; Formal analysis: EPKP; Writing–original draft: EPKP; Writing–review & editing: EPKP, TH, LN, GA, NA, EVHB, IJD, KEM, AS, ML, BM, WJH, LAT; Visualisation: EPKP; Project administration: EPKP, WJH, LAT; Funding acquisition: EPKP, LAT. All authors had access to the aggregated data included in the manuscript. All authors contributed to and approved the final manuscript.

## Data sharing statement

Detailed pseudonymised patient data are potentially re-identifiable and therefore not shared. Access to the underlying identifiable and potentially re-identifiable pseudonymised electronic health record data is tightly governed by various legislative and regulatory frameworks, and restricted by best practice. The data in OpenSAFELY is drawn from General Practice data across England where TPP is the data processor. TPP developers initiate an automated process to create pseudonymised records in the core OpenSAFELY database, which are copies of key structured data tables in the identifiable records. These pseudonymised records are linked onto key external data resources that have also been pseudonymised via SHA-512 one-way hashing of NHS numbers using a shared salt. Bennett Institute for Applied Data Science developers and PIs holding contracts with NHS England have access to the OpenSAFELY pseudonymised data tables as needed to develop the OpenSAFELY tools. These tools in turn enable researchers with OpenSAFELY data access agreements to write and execute code for data management and data analysis without direct access to the underlying raw pseudonymised patient data, and to review the outputs of this code. All code for the full data management pipeline – from raw data to completed results for this analysis – and for the OpenSAFELY platform as a whole is available for review at github.com/OpenSAFELY.

## Information governance

NHS England is the data controller for the NHS England OpenSAFELY COVID-19 Service; TPP is the data processor; all study authors using OpenSAFELY have the approval of NHS England. This implementation of OpenSAFELY is hosted within the TPP environment which is accredited to the ISO 27001 information security standard and is NHS IG Toolkit compliant.

Patient data has been pseudonymised for analysis and linkage using industry standard cryptographic hashing techniques; all pseudonymised datasets transmitted for linkage onto OpenSAFELY are encrypted; access to the NHS England OpenSAFELY COVID-19 service is via a virtual private network (VPN) connection; the researchers hold contracts with NHS England and only access the platform to initiate database queries and statistical models; all database activity is logged; only aggregate statistical outputs leave the platform environment following best practice for anonymisation of results such as statistical disclosure control for low cell counts.

The service adheres to the obligations of the UK General Data Protection Regulation (UK GDPR) and the Data Protection Act 2018. The service previously operated under notices initially issued in February 2020 by the Secretary of State under Regulation 3(4) of the Health Service (Control of Patient Information) Regulations 2002 (COPI Regulations), which required organisations to process confidential patient information for COVID-19 purposes; this set aside the requirement for patient consent. As of 1 July 2023, the Secretary of State has requested that NHS England continue to operate the Service under the COVID-19 Directions 2020. In some cases of data sharing, the common law duty of confidence is met using, for example, patient consent or support from the Health Research Authority Confidentiality Advisory Group.

Taken together, these provide the legal bases to link patient datasets using the service. GP practices, which provide access to the primary care data, are required to share relevant health information to support the public health response to the pandemic, and have been informed of how the service operates.

## Code availability

Data management was performed using Python, with analysis carried out using R 4.0.2. Code for data management and analysis, as well as codelists, are archived online (https://github.com/opensafely/covid-risk-immunocompromised).

## Declaration of interests

EPKP has received funding from NIHR (NIHR206900, NIHR209347) and Bill & Melinda Gates Foundation (OPP1210509). He has received funding as a consultant for Tulane University as part of the ‘Safe in Pregnancy and Childhood Study’, supported by the Safety Platform for Emergency vACcines (SPEAC), Task Force for Global Health, Inc. – The Coalition for Epidemic Preparedness Innovations (CEPI). He received reimbursements for providing input on an expert report for the UK COVID-19 Inquiry. LN is currently a salaried employee at University Medical Center Utrecht, which receives institutional research funding from pharmaceutical companies and regulatory agencies, administered by University Medical Center Utrecht. All these studies follow the ENCePP code of conduct. These funds may also be used to support attendance at meetings and related travel expenses. ID has received unrelated research grants from GlaxoSmithKline. He holds shares in GlaxoSmithKline. KEM declares personal consulting fees from AMGEN outside of the submitted work. AS has received funding for research from Open Philanthropy (now Coefficient Giving) and Alzheimer’s Research UK (ARUK-SPON2025-005). She has received payment as an external course lecturer. ML is currently employed at Invivyd, Inc. Her academic contribution to this work was provided while she was affiliated with the University of Oxford. The authors disclose no other competing interests. LAT has received travel expenses for MHRA expert advisory group meetings. She is an unpaid member of three trial steering committees (NIHR funded).
