# Supplementary material for: Factors associated with severe COVID-19 in immunocompromised subgroups in England from 2020 to 2024: an OpenSAFELY cohort study
Source: eBioMedicine. 2026 Jun 26;129:106327. doi: 10.1016/j.ebiom.2026.106327 (PMC13324492; doi:10.1016/j.ebiom.2026.106327)
Supplement: Supplementary Figs. S1–S11 and Tables S1–S7 [file mmc3.docx]

**Supplementary Appendix**

Supplement to: Parker EPK, Hartney T, Nab L, et al. Factors associated with severe COVID-19 in immunocompromised subgroups in England from 2020 to 2024: an OpenSAFELY cohort study

Corresponding author: edward.parker@lshtm.ac.uk

**Contents:**

P2: Supplementary Tables 1–7

P13: Supplementary Figures 1–11

P24: RECORD checklist

**Table S1. Definition of key variables.**

| **Variable** | **Notes** | **Values** | **Codelist ID** |
| --- | --- | --- | --- |
| **Index dates (start of wave)** |  | Wave 1: 23 March 2020  Alpha: 07 September 2020  Delta: 28 May 2021  Omicron BA.1/BA.2: 15 December 2021  JN.1: 04 December 2023 |  |
| **End date** | – | Wave 1: 30 May 2020  Alpha: 24 April 2021  Delta: 14 December 2021  Omicron BA.1/BA.2: 29 April 2022  JN.1: 31 March 2024 |  |
| **Demography** |  |  |  |
| Age group (years) | Age in years at index date | 18–39; 40–49; 50–59; 60–69; 70–79; 80+ | – |
| Sex | – | Female; Male | – |
| Ethnicity | Derived from most recent record in primary care or (if missing) Secondary Use Service | White; Black; South Asian; Other | opensafely/ethnicity/2020-04-27 |
| Region | NHS region derived from practice address | North East; North West; Yorkshire and the Humber; East Midlands; West Midlands; East of England; London; South East; South West | – |
| Index of multiple deprivation | Social deprivation quintile derived from individual’s address at Lower Super Output Area (a small geographical area defined by the ONS) | 1 (most deprived); 2; 3; 4; 5 (least deprived) | – |
| Care home | Based on matching of individual’s address with care homes in CQC database or presence of code in primary record before date defined | 0; 1 | primis-covid19-vacc-uptake/longres/v2.5 |
| Smoking status | Derived from most recent record in primary care | Never and unknown; Former; Current | opensafely/smoking-clear/2020-04-29 |
| **Immunocompromise status** |  |  |  |
| Solid organ transplant (SOT) | Any prior code in primary care record | 0; 1 (and date of most recent record) | opensafely/kidney-transplant/2020-07-15  opensafely/other-organ-transplant/79caeeee/ |
| Bone marrow compromise (BMC) | Any prior code in primary care record | 0; 1 (and date of most recent record) | opensafely/bone-marrow-transplant/2020-04-15;  opensafely/haematological-cancer/2020-04-15 |
| Radio-/chemo-therapy (RCT) | Any code in primary care record in the 6 months preceding index date | 0; 1 | primis-covid19-vacc-uptake/dxt_chemo_cod/v2.5 |
| Immunosuppressive medication (IMM) | Any code in primary care record in the 6 months preceding index date | 0; 1 | primis-covid19-vacc-uptake/immrx/v2.5 |
| Primary or acquired immunodeficiency (IMD) | Any prior code in primary care record | 0; 1 (and date of most recent record) | primis-covid19-vacc-uptake/immdx_cov/v2.5 |
| Immunocompromised subgroup | Hierarchical assignment to the five subgroups above | SOT; BMC; RCT; IMM; IMD | – |
| **Type of immunocompromise** | Defined for SOT based on presence of kidney vs non-kidney transplant and for BMC based on bone marrow transplant vs no bone marrow transplant | SOT: kidney; other  BMC: bone marrow transplant; no transplant | – |
| **Timing of immunocompromise** | Date of most recent record | ≤1 year; > 1 year (SOT, BMC, IMD) | – |
| **COVID-19 vaccination history** |  |  |  |
| N primary doses | COVID-19 vaccination records in the National Immunisation Management System (later replaced by the National Immunisation and Vaccination System) are transferred to an individual’s primary care record within days. Sequential vaccination dates were extracted for any COVID-19 vaccine, with any records with an interval of ≥14 days considered separate doses. | - Delta: 0; 1; 2 - Omicron: 0; 1; 2; 3+ - JN.1: 0–4; 5–6; 7+ | – |
| Timing of most recent dose | Defined based on date of most recent vaccine record for any COVID-19 vaccine product. | 27+ weeks or unvaccinated; 13–26 weeks; 0–12 weeks |  |
| **Prior infection status** | Defined based on the date of the most recent prior record of a positive SARS-CoV-2 test, COVID-19-related hospitalisation (as defined below), COVID-19-related A&E attendance (defined using the SNOMED code 1240751000000100), or (in wave 1) probable COVID-19 based on primary care coding.  Episodes were assigned to waves as follows:   - Wave 1: 23/03/2020 to 06/09/2020 - Alpha: 06/09/2020 to 27/05/2021 - Delta: 28/05/2021 to 14/12/2021 - Omicron BA.1/BA.2: 15/12/2021 to 05/06/2022 - Omicron BA.5/XBB: 06/06/2022 to 03/12/2023^a^ | - Alpha: No prior infection; Infected (wave 1) - Delta: No prior infection; Infected (wave 1); Infected (alpha) - BA.1/BA.2: No prior infection; Infected (pre-Delta); Infected (Delta) - JN>1: No prior infection; Infected (pre-Omicron); Infected (Omicron) | opensafely/covid-identification-in-primary-care-probable-covid-clinical-code/2020-07-16;  opensafely/covid-identification-in-primary-care-probable-covid-positive-test/2020-07-16;  opensafely/covid-identification-in-primary-care-probable-covid-sequelae/2020-07-16 |
| **Other clinical risk groups** |  |  |  |
| Body mass index | Derived from most recent body mass index record in the 5 years preceding index date either computed from weight and height measurements or, where they are not available, from recorded body mass index values | Not obese; Obese I (30–34.9 kg/m^2^); Obese II (35–39.9 kg/m^2^); Obese III (40+ kg/m^2^) | – |
| Asthma | Asthma was defined based on a primary care diagnosis in the 3 years preceding index date or any prior diagnosis in the absence of another chronic respiratory disease diagnosis. Oral steroid use was defined based on oral prednisolone prescriptions in the year preceding index date. | No asthma; With no oral steroid use; With oral steroid use | opensafely/asthma-diagnosis/2020-04-15;  opensafely/asthma-oral-prednisolone-medication/2020-04-27 |
| Diabetes mellitus | Diabetes was defined based on any prior primary care diagnosis. Controlled vs uncontrolled diabetes was defined based on the most recent Hb1ac measure in the 15 months preceding index date, with a level of <7.5% (or <58 mmol/mol) used to define controlled diabetes. | No diabetes; Controlled; Not controlled; Without recent Hb1ac measure | opensafely/diabetes/2020-04-15;  opensafely/glycated-haemoglobin-hba1c-tests-ifcc/6d7287f8 |
| CKD or RRT | Most recent serum creatinine level (in μmol/l) and age on date of creatinine measurement were used to calculate estimated glomerular filtrate rate (eGFR), then converted to CKD status as defined below. Most recent RRT status (dialysis or transplant) superseded CKD status where applicable.  eGFR thresholds were as follows:   - Stage 3a: 45–59 mL/min/1.73 m^2^ - Stage 3b: 30–44 mL/min/1.73 m^2^ - Stage 4–5: 15–29 mL/min/1.73 m^2^ - Stage 5L <30 mL/min/1.73 m^2^ | No CKD or RRT; CKD stage 3a; CKD stage 3b; CKD stage 4; CKD stage 5; RRT (dialysis); RRT (transplant) | opensafely/kidney-transplant/2020-07-15;  opensafely/dialysis/3ce108ac |
| Hypertension | High blood pressure (systolic blood pressure ≥140 or diastolic blood pressure ≥90) or any prior primary care diagnosis of hypertension | 0; 1 | opensafely/hypertension/2020-04-28 |
| Chronic respiratory disease | Any prior code in primary care record | 0; 1 | opensafely/chronic-respiratory-disease/2020-04-10 |
| Chronic cardiac disease | Any prior code in primary care record | 0; 1 | opensafely/chronic-cardiac-disease/2020-04-08 |
| Cancer (non haematological) | Any prior code in primary care record | 0; 1 | opensafely/lung-cancer/2020-04-15;  opensafely/cancer-excluding-lung-and-haematological/2020-04-15 |
| Chronic liver disease | Any prior code in primary care record | 0; 1 | opensafely/chronic-liver-disease/2020-06-02 |
| Stroke | Any prior code in primary care record | 0; 1 | opensafely/stroke-updated/2020-06-02 |
| Dementia | Any prior code in primary care record | 0; 1 | opensafely/dementia/2020-04-22 |
| Other neurological disease | Any prior code in primary care record | 0; 1 | opensafely/other-neurological-conditions/2020-06-02 |
| Asplenia | Any prior code in primary care record | 0; 1 | opensafely/asplenia/2020-06-02;  opensafely/sickle-cell-disease/2020-04-14 |
| Rheumatoid arthritis/lupus/psoriasis | Any prior code in primary care record | 0; 1 | opensafely/ra-sle-psoriasis/2020-04-14 |
| Learning disability | Any prior code in primary care record | 0; 1 | nhsd-primary-care-domain-refsets/ld_cod/20210127 |
| Severe mental illness | Any prior code in primary care record | 0; 1 | primis-covid19-vacc-uptake/sev_mental/v2.5 |
| Severe obesity | Most recent body mass index of ≥40 kg/m^2^ | 0; 1 | – |
| **Outcomes** |  |  |  |
| Severe COVID-19 | Defined based on a composite of:   - Hospital episodes with ICD-10 diagnosis codes of U07.1 (“covid-19, virus identified”) or U07.2 (“covid-19, virus not identified”)^b^ as the primary or non-primary reason for admission and with an admission method in the following list: ”21”, ”22”, ”23”, ”24”, ”25”, ”2A”, ”2B”, ”2C”, ”2D”, ”28” - Accident and emergency department attendances with a COVID-19 SNOMED diagnosis code (“1240751000000100”) - COVID-19-related death (as defined below) | Date | – |
| COVID-19-related death | Defined based on death certificates with ICD-10 codes of U07.1 or U07.1 as an underlying or contributing cause | Date | – |
| Deregistration | Used for censoring | Date | – |
| Non-COVID-19 death | Death from any cause as registered in ONS, excluding COVID-19-related deaths as defined above | Date | – |

Additional details on variable coding can be found in the full analysis scripts (available at https://github.com/opensafely/ckd-coverage-ve). Codelists can be found at https://codelists.opensafely.org/codelist/[Codelist ID], substituting [Codelist ID] with the identifier listed above. CQC, Care and Quality Commission; CKD, chronic kidney disease; CTV3, Clinical Terms Version 3; ICD-10, international statistical classification of diseases, 10th revision; JCVI, Joint Committee on Vaccination and Immunisation; ONS, Office for National Statistics; NIMS; National Immunisation Management Service; RRT, renal replacement therapy; SGSS, Second Generation Surveillance System.

^a^ Omicron transitions reflect dates in which variants became nationally dominant in UK Health Security Agency genomic surveillance records.

^b^ Used “when COVID-19 is diagnosed clinically or epidemiologically but laboratory testing is inconclusive or not available” (International Statistical Classification of Diseases and Related Health Problems 10th Revision).

##### Table S2. Cohort selection.

|  | **N retained (%)** | | | | |
| --- | --- | --- | --- | --- | --- |
| **Criteria** | **Wave 1** | **Alpha** | **Delta** | **BA.1/BA.2** | **JN.1** |
| Registered for at least 3 months, aged ≥18 years, male or female, and at least one indicator of immunocompromise on index | 482,270 | 481,180 | 486,760 | 492,210 | 515,335 |
| No missing demographic information (region, index of multiple deprivation) | 482,030 (100) | 480,940 (100) | 486,520 (100) | 491,940 (99.9) | 513,885 (99.7) |
| No outcome or censoring event (death or deregistration) on index date | 481,990 (99.9) | 480,935 (99.9) | 486,515 (99.9) | 491,890 (99.9) | 513,860 (99.7) |
| No documented SARS-CoV-2 infection in 90 days before index date | 481,990 (99.9) | 479,485 (99.6) | 484,565 (99.5) | 475,360 (96.6) | 508,545 (98.7) |

Counts are rounded to the nearest 5.

##### Table S3. Intersections of immunocompromise coding in primary care.

| **Criteria** | **N** |
| --- | --- |
| **Subgroup size (prior to hierarchical assignment)** |  |
| SOT | 20,070 |
| BMC | 113,290 |
| RCT | 13,190 |
| IMM | 150,905 |
| IMD | 373,895 |
| **Intersection size** |  |
| IMD alone | 189,770 |
| IMD + BMC | 106,695 |
| IMM alone | 71,390 |
| IMM + IMD | 70,415 |
| SOT alone | 11,740 |
| RCT alone | 10,465 |
| SOT + IMM | 5,935 |
| BMC + RCT + IMD | 2,065 |
| BMC + IMM + IMD | 2,060 |
| BMC alone | 1,695 |
| SOT + IMD | 1,260 |
| SOT + IMM + IMD | 535 |
| Other (sum of intersections with N < 500) | 1,340 |

Data shown for the start of the Omicron BA.1/BA.2 wave (15 December 2021). Counts are rounded to the nearest 5. BMC, bone marrow compromising condition; IMD, primary or acquired immunodeficiency; IMM, immunosuppressive medication in past 6 months; RCT, radiotherapy or chemotherapy in past 6 months; SOT, solid organ transplant.

**Table S4. Baseline characteristics across waves.**

| **Variable** | **Level** | **Wave 1**  **(N = 481,990)** | **Alpha**  **(N = 479,485)** | **Delta**  **(N = 484,565)** | **BA.1/BA.2**  **(N = 475,360)** | **JN.1**  **(N = 508,545)** |
| --- | --- | --- | --- | --- | --- | --- |
| Age group (years) | 18-39 | 57,125 (11.9%) | 56,400 (11.8%) | 56,655 (11.7%) | 53,575 (11.3%) | 56,415 (11.1%) |
|  | 40-49 | 55,480 (11.5%) | 54,550 (11.4%) | 54,350 (11.2%) | 51,165 (10.8%) | 54,425 (10.7%) |
|  | 50-59 | 89,475 (18.6%) | 88,990 (18.6%) | 89,955 (18.6%) | 87,540 (18.4%) | 91,690 (18.0%) |
|  | 60-69 | 100,935 (20.9%) | 100,490 (21.0%) | 102,085 (21.1%) | 101,045 (21.3%) | 110,610 (21.8%) |
|  | 70-79 | 110,340 (22.9%) | 110,215 (23.0%) | 112,890 (23.3%) | 112,850 (23.7%) | 119,405 (23.5%) |
|  | 80+ | 68,630 (14.2%) | 68,845 (14.4%) | 68,635 (14.2%) | 69,185 (14.6%) | 76,000 (14.9%) |
| Sex | Female | 274,020 (56.9%) | 272,730 (56.9%) | 275,840 (56.9%) | 270,585 (56.9%) | 289,890 (57.0%) |
|  | Male | 207,970 (43.1%) | 206,755 (43.1%) | 208,730 (43.1%) | 204,775 (43.1%) | 218,655 (43.0%) |
| Ethnicity | White | 422,595 (87.7%) | 419,885 (87.6%) | 424,950 (87.7%) | 416,235 (87.6%) | 442,450 (87.0%) |
|  | South Asian | 24,655 (5.1%) | 24,745 (5.2%) | 25,175 (5.2%) | 25,365 (5.3%) | 29,260 (5.8%) |
|  | Black | 15,820 (3.3%) | 15,870 (3.3%) | 16,080 (3.3%) | 16,140 (3.4%) | 18,905 (3.7%) |
|  | Other | 18,920 (3.9%) | 18,980 (4.0%) | 18,365 (3.8%) | 17,625 (3.7%) | 17,930 (3.5%) |
| Region | North East | 22,875 (4.7%) | 22,700 (4.7%) | 23,015 (4.7%) | 22,405 (4.7%) | 23,805 (4.7%) |
|  | North West | 43,265 (9.0%) | 42,735 (8.9%) | 43,140 (8.9%) | 42,335 (8.9%) | 45,680 (9.0%) |
|  | Yorkshire and the Humber | 67,020 (13.9%) | 66,520 (13.9%) | 67,115 (13.9%) | 66,195 (13.9%) | 70,805 (13.9%) |
|  | East Midlands | 81,660 (16.9%) | 81,320 (17.0%) | 82,030 (16.9%) | 80,575 (17.0%) | 86,770 (17.1%) |
|  | West Midlands | 17,550 (3.6%) | 17,410 (3.6%) | 17,310 (3.6%) | 17,075 (3.6%) | 18,110 (3.6%) |
|  | East of England | 115,415 (23.9%) | 115,095 (24.0%) | 116,520 (24.0%) | 113,715 (23.9%) | 120,975 (23.8%) |
|  | London | 26,445 (5.5%) | 26,410 (5.5%) | 26,610 (5.5%) | 26,395 (5.6%) | 28,080 (5.5%) |
|  | South East | 33,510 (7.0%) | 33,190 (6.9%) | 33,580 (6.9%) | 32,845 (6.9%) | 35,060 (6.9%) |
|  | South West | 74,245 (15.4%) | 74,110 (15.5%) | 75,245 (15.5%) | 73,825 (15.5%) | 79,250 (15.6%) |
| Deprivation quintile | 5 (least deprived) | 95,095 (19.7%) | 94,630 (19.7%) | 96,060 (19.8%) | 94,070 (19.8%) | 100,720 (19.8%) |
|  | 4 | 103,660 (21.5%) | 103,380 (21.6%) | 104,680 (21.6%) | 102,805 (21.6%) | 110,080 (21.6%) |
|  | 3 | 105,920 (22.0%) | 105,420 (22.0%) | 106,660 (22.0%) | 104,815 (22.0%) | 112,290 (22.1%) |
|  | 2 | 92,580 (19.2%) | 92,035 (19.2%) | 92,805 (19.2%) | 90,885 (19.1%) | 97,295 (19.1%) |
|  | 1 (most deprived) | 84,735 (17.6%) | 84,020 (17.5%) | 84,360 (17.4%) | 82,790 (17.4%) | 88,160 (17.3%) |
| Care home |  | 6,565 (1.4%) | 6,180 (1.3%) | 6,305 (1.3%) | 6,490 (1.4%) | 7,730 (1.5%) |
| Smoking status | Never and unknown | 201,015 (41.7%) | 200,395 (41.8%) | 202,445 (41.8%) | 198,455 (41.7%) | 213,865 (42.1%) |
|  | Former | 219,785 (45.6%) | 218,675 (45.6%) | 221,710 (45.8%) | 217,740 (45.8%) | 235,465 (46.3%) |
|  | Current | 61,185 (12.7%) | 60,420 (12.6%) | 60,410 (12.5%) | 59,165 (12.4%) | 59,210 (11.6%) |
| Immunocompromised subgroup | SOT | 20,885 (4.3%) | 20,875 (4.4%) | 20,925 (4.3%) | 20,070 (4.2%) | 21,350 (4.2%) |
|  | BMC | 112,475 (23.3%) | 112,820 (23.5%) | 114,205 (23.6%) | 112,710 (23.7%) | 120,300 (23.7%) |
|  | RCT | 11,455 (2.4%) | 9,760 (2.0%) | 10,825 (2.2%) | 11,005 (2.3%) | 12,615 (2.5%) |
|  | IMM | 148,410 (30.8%) | 145,625 (30.4%) | 145,950 (30.1%) | 141,805 (29.8%) | 148,815 (29.3%) |
|  | IMD | 188,760 (39.2%) | 190,405 (39.7%) | 192,660 (39.8%) | 189,770 (39.9%) | 205,465 (40.4%) |
| N prior COVID-19 vaccine doses (Delta, BA.1/BA.2) | 0 | – | – | 32,710 (6.8%) | 24,175 (5.1%) | – |
|  | 1 | – | – | 39,910 (8.2%) | 7,365 (1.5%) | – |
|  | 2 | – | – | 411,945 (85.0%) | 69,300 (14.6%) | – |
|  | 3+ | – | – | – | 374,525 (78.8%) | – |
| N prior COVID-19 vaccine doses (JN.1) | 0-4 | – | – | – | – | 193,330 (38.0%) |
|  | 5–6 | – | – | – | – | 152,295 (29.9%) |
|  | 7+ | – | – | – | – | 162,920 (32.0%) |
| Timing of last COVID-19 vaccine dose | 27+ weeks/unvaccinated | – | – | 32,710 (6.8%) | 78,360 (16.5%) | 206,085 (40.5%) |
|  | 13-26 weeks | – | – | 23,310 (4.8%) | 17,620 (3.7%) | 8,125 (1.6%) |
|  | 0-12 weeks | – | – | 428,545 (88.4%) | 379,380 (79.8%) | 294,330 (57.9%) |
| Prior infection | No prior infection | – | 475,355 (99.1%) | 458,985 (94.7%) | 437,050 (91.9%) | 346,495 (68.1%) |
|  | Infected (WT) | – | 4,130 (0.9%) | 4,865 (1.0%) | – | – |
|  | Infected (Alpha) | – | – | 20,720 (4.3%) | – | – |
|  | Infected (Pre Delta) | – | – | – | 26,715 (5.6%) | – |
|  | Infected (Delta) | – | – | – | 11,600 (2.4%) | – |
|  | Infected (Pre Omicron) | – | – | – | – | 45,920 (9.0%) |
|  | Infected (BA.1/BA.2) | – | – | – | – | 68,240 (13.4%) |
|  | Infected (BA.5/XBB) | – | – | – | – | 47,885 (9.4%) |
| Comorbidity count | 0 | 118,340 (24.6%) | 118,085 (24.6%) | 118,715 (24.5%) | 114,695 (24.1%) | 119,425 (23.5%) |
|  | 1 | 163,705 (34.0%) | 162,565 (33.9%) | 163,105 (33.7%) | 158,985 (33.4%) | 165,200 (32.5%) |
|  | 2 | 121,635 (25.2%) | 120,765 (25.2%) | 121,770 (25.1%) | 119,990 (25.2%) | 129,535 (25.5%) |
|  | 3 | 58,100 (12.1%) | 57,950 (12.1%) | 59,750 (12.3%) | 59,980 (12.6%) | 68,340 (13.4%) |
|  | 4+ | 20,210 (4.2%) | 20,120 (4.2%) | 21,230 (4.4%) | 21,710 (4.6%) | 26,040 (5.1%) |
| Body mass index | Not obese | 350,125 (72.6%) | 348,115 (72.6%) | 351,290 (72.5%) | 345,085 (72.6%) | 369,520 (72.7%) |
|  | Obese I (30-34.9 kg/m^2^) | 79,255 (16.4%) | 78,870 (16.4%) | 79,800 (16.5%) | 78,035 (16.4%) | 82,770 (16.3%) |
|  | Obese II (35-39.9 kg/m^2^) | 32,820 (6.8%) | 32,675 (6.8%) | 33,260 (6.9%) | 32,480 (6.8%) | 34,920 (6.9%) |
|  | Obese III (40+ kg/m^2^) | 19,790 (4.1%) | 19,825 (4.1%) | 20,215 (4.2%) | 19,760 (4.2%) | 21,330 (4.2%) |
| Asthma | No asthma | 394,320 (81.8%) | 391,770 (81.7%) | 395,545 (81.6%) | 388,040 (81.6%) | 413,350 (81.3%) |
|  | With no oral steroid use | 72,000 (14.9%) | 73,000 (15.2%) | 77,845 (16.1%) | 74,610 (15.7%) | 77,760 (15.3%) |
|  | With oral steroid use | 15,665 (3.3%) | 14,715 (3.1%) | 11,175 (2.3%) | 12,710 (2.7%) | 17,430 (3.4%) |
| Diabetes | No diabetes | 391,035 (81.1%) | 388,225 (81.0%) | 388,710 (80.2%) | 377,450 (79.4%) | 387,955 (76.3%) |
|  | Controlled | 78,100 (16.2%) | 74,975 (15.6%) | 76,510 (15.8%) | 82,115 (17.3%) | 107,670 (21.2%) |
|  | Not controlled | 3,490 (0.7%) | 3,295 (0.7%) | 3,235 (0.7%) | 3,035 (0.6%) | 2,650 (0.5%) |
|  | Without recent Hb1ac measure | 9,360 (1.9%) | 12,990 (2.7%) | 16,110 (3.3%) | 12,765 (2.7%) | 10,265 (2.0%) |
| CKD or RRT | No CKD or RRT | 400,840 (83.2%) | 398,785 (83.2%) | 402,490 (83.1%) | 393,810 (82.8%) | 419,495 (82.5%) |
|  | CKD stage 3a | 41,835 (8.7%) | 41,600 (8.7%) | 42,400 (8.8%) | 42,435 (8.9%) | 46,995 (9.2%) |
|  | CKD stage 3b | 17,415 (3.6%) | 17,350 (3.6%) | 17,850 (3.7%) | 17,820 (3.7%) | 19,155 (3.8%) |
|  | CKD stage 4 | 4,745 (1.0%) | 4,640 (1.0%) | 4,665 (1.0%) | 4,820 (1.0%) | 5,200 (1.0%) |
|  | CKD stage 5 | 570 (0.1%) | 550 (0.1%) | 585 (0.1%) | 575 (0.1%) | 660 (0.1%) |
|  | RRT (dialysis) | 3,660 (0.8%) | 3,715 (0.8%) | 3,745 (0.8%) | 3,670 (0.8%) | 4,110 (0.8%) |
|  | RRT (transplant) | 12,925 (2.7%) | 12,845 (2.7%) | 12,835 (2.6%) | 12,235 (2.6%) | 12,930 (2.5%) |
| Other clinical risk group | Hypertension | 259,970 (53.9%) | 257,665 (53.7%) | 261,805 (54.0%) | 258,255 (54.3%) | 276,070 (54.3%) |
|  | Chronic respiratory disease | 54,895 (11.4%) | 53,980 (11.3%) | 54,225 (11.2%) | 53,540 (11.3%) | 57,865 (11.4%) |
|  | Chronic cardiac disease | 74,575 (15.5%) | 73,725 (15.4%) | 74,485 (15.4%) | 74,115 (15.6%) | 80,220 (15.8%) |
|  | Cancer (non-haematological) | 59,870 (12.4%) | 58,495 (12.2%) | 60,640 (12.5%) | 61,175 (12.9%) | 68,345 (13.4%) |
|  | Chronic liver disease | 14,460 (3.0%) | 14,525 (3.0%) | 15,005 (3.1%) | 14,875 (3.1%) | 16,735 (3.3%) |
|  | Stroke | 23,550 (4.9%) | 23,320 (4.9%) | 23,730 (4.9%) | 23,880 (5.0%) | 26,885 (5.3%) |
|  | Dementia | 2,570 (0.5%) | 2,350 (0.5%) | 2,285 (0.5%) | 2,260 (0.5%) | 2,245 (0.4%) |
|  | Other neurological disease | 15,125 (3.1%) | 15,020 (3.1%) | 14,995 (3.1%) | 14,855 (3.1%) | 15,930 (3.1%) |
|  | Asplenia | 4,395 (0.9%) | 4,285 (0.9%) | 4,275 (0.9%) | 4,205 (0.9%) | 4,325 (0.9%) |
|  | Rheumatoid arthritis/lupus/psoriasis | 212,640 (44.1%) | 211,945 (44.2%) | 213,635 (44.1%) | 209,160 (44.0%) | 222,425 (43.7%) |
|  | Learning disability | 2,430 (0.5%) | 2,435 (0.5%) | 2,510 (0.5%) | 2,510 (0.5%) | 2,900 (0.6%) |
|  | Severe mental illness | 6,565 (1.4%) | 6,620 (1.4%) | 6,770 (1.4%) | 6,745 (1.4%) | 7,385 (1.5%) |

Results are aggregated across immunocompromised subgroups. BMC, bone marrow compromising condition; CKD, chronic kidney disease; IMD, primary or acquired immunodeficiency; IMM, immunosuppressive medication in past 6 months; RCT, radiotherapy or chemotherapy in past 6 months; RRT, renal replacement therapy; SOT, solid organ transplant.

**Table S5. Absolute rates of severe COVID-19 outcomes across waves.**

| **Outcome** | **Wave** | **Metric** | **SOT** | **BMC** | **RCT** | **IMM** | **IMD** |
| --- | --- | --- | --- | --- | --- | --- | --- |
| Severe COVID-19 | Wave 1 | N | 20,885 | 112,475 | 11,455 | 148,410 | 188,760 |
|  |  | N events | 265 | 1,100 | 160 | 840 | 1,405 |
|  |  | Person-time | 1,402,295 | 7,547,615 | 752,430 | 10,034,395 | 12,717,460 |
|  |  | IR per 1000 person-years (95% CI) | 69 (60.7-77.3) | 53.2 (50.1-56.4) | 77.7 (65.6-89.7) | 30.6 (28.5-32.6) | 40.4 (38.2-42.5) |
|  | Alpha | N | 20,875 | 112,820 | 9760 | 145,625 | 190,405 |
|  |  | N events | 600 | 2285 | 265 | 2045 | 3165 |
|  |  | Person-time | 4,649,325 | 25,008,580 | 1,982,795 | 32,822,360 | 42,560,990 |
|  |  | IR per 1000 person-years (95% CI) | 47.1 (43.4-50.9) | 33.4 (32-34.7) | 48.8 (42.9-54.7) | 22.8 (21.8-23.7) | 27.2 (26.2-28.1) |
|  |  | IRR relative to wave 1 | 0.68 | 0.63 | 0.63 | 0.75 | 0.67 |
|  | Delta | N | 20,925 | 114,205 | 10,825 | 145,950 | 192,660 |
|  |  | N events | 665 | 1,415 | 125 | 1,060 | 1,445 |
|  |  | Person-time | 4,081,110 | 22,297,540 | 1,987,540 | 28,862,015 | 37,882,610 |
|  |  | IR per 1000 person-years (95% CI) | 59.5 (55-64) | 23.2 (22-24.4) | 23 (18.9-27) | 13.4 (12.6-14.2) | 13.9 (13.2-14.7) |
|  |  | IRR relative to wave 1 | 0.86 | 0.44 | 0.30 | 0.44 | 0.34 |
|  | Omicron (BA.1/BA.2) | N | 20,070 | 112,710 | 11,005 | 141,805 | 189,770 |
|  |  | N events | 825 | 2,145 | 295 | 1,610 | 2,205 |
|  |  | Person-time | 2,632,910 | 14,876,240 | 1,399,730 | 18,927,960 | 25,232,480 |
|  |  | IR per 1000 person-years (95% CI) | 114.4 (106.6-122.3) | 52.7 (50.4-54.9) | 77 (68.2-85.8) | 31.1 (29.6-32.6) | 31.9 (30.6-33.3) |
|  |  | IRR relative to wave 1 | 1.66 | 0.99 | 0.99 | 1.02 | 0.79 |
|  | Omicron (JN.1) | N | 21,350 | 120,300 | 12,615 | 148,815 | 205,465 |
|  |  | N events | 140 | 600 | 115 | 310 | 495 |
|  |  | Person-time | 2,485,370 | 13,979,915 | 1,420,255 | 17,451,475 | 23,981,790 |
|  |  | IR per 1000 person-years (95% CI) | 20.6 (17.2-24) | 15.7 (14.4-16.9) | 29.6 (24.2-35) | 6.5 (5.8-7.2) | 7.5 (6.9-8.2) |
|  |  | IRR relative to wave 1 | 0.30 | 0.30 | 0.38 | 0.21 | 0.19 |
| COVID-19-related mortality | Wave 1 | N | 20,885 | 112,475 | 11,455 | 148,410 | 188,760 |
|  |  | N events | 85 | 480 | 45 | 280 | 585 |
|  |  | Person-time | 1,410,520 | 7,573,465 | 756,685 | 10,057,345 | 12,750,975 |
|  |  | IR per 1000 person-years (95% CI) | 22 (17.3-26.7) | 23.1 (21.1-25.2) | 21.7 (15.4-28.1) | 10.2 (9-11.4) | 16.8 (15.4-18.1) |
|  | Alpha | N | 20,875 | 112,820 | 9760 | 145,625 | 190,405 |
|  |  | N events | 190 | 945 | 90 | 610 | 1170 |
|  |  | Person-time | 4,699,345 | 25,169,710 | 2,001,110 | 32,995,455 | 42,800,665 |
|  |  | IR per 1000 person-years (95% CI) | 14.8 (12.7-16.9) | 13.7 (12.8-14.6) | 16.4 (13-19.8) | 6.8 (6.2-7.3) | 10 (9.4-10.6) |
|  |  | IRR relative to wave 1 | 0.67 | 0.59 | 0.76 | 0.67 | 0.60 |
|  | Delta | N | 20,925 | 114,205 | 10,825 | 145,950 | 192,660 |
|  |  | N events | 170 | 380 | 30 | 225 | 335 |
|  |  | Person-time | 4,121,505 | 22,377,515 | 1,995,265 | 28,927,270 | 37,968,345 |
|  |  | IR per 1000 person-years (95% CI) | 15.1 (12.8-17.3) | 6.2 (5.6-6.8) | 5.5 (3.5-7.5) | 2.8 (2.5-3.2) | 3.2 (2.9-3.6) |
|  |  | IRR relative to wave 1 | 0.69 | 0.27 | 0.25 | 0.28 | 0.19 |
|  | Omicron (BA.1/BA.2) | N | 20,070 | 112,710 | 11,005 | 141,805 | 189,770 |
|  |  | N events | 75 | 355 | 35 | 145 | 270 |
|  |  | Person-time | 2,680,930 | 14,990,845 | 1,415,660 | 19,020,915 | 25,355,575 |
|  |  | IR per 1000 person-years (95% CI) | 10.2 (7.9-12.5) | 8.6 (7.7-9.5) | 9 (6-12) | 2.8 (2.3-3.2) | 3.9 (3.4-4.4) |
|  |  | IRR relative to wave 1 | 0.46 | 0.37 | 0.42 | 0.28 | 0.23 |
|  | Omicron (JN.1) | N | 21,350 | 120,300 | 12,615 | 148,815 | 205,465 |
|  |  | N events | [R] | 80 | [R] | 30 | 60 |
|  |  | Person-time | [R] | 14,018,495 | [R] | 17,472,985 | 24,014,330 |
|  |  | IR per 1000 person-years (95% CI) | [R] | 2.1 (1.6-2.5) | [R] | 0.6 (0.4-0.9) | 0.9 (0.7-1.1) |
|  |  | IRR relative to wave 1 | [R] | 0.09 | [R] | 0.06 | 0.05 |

BMC, bone marrow compromising condition; IMD, primary or acquired immunodeficiency; IMM, immunosuppressive medication in past 6 months; IR, incidence rate; [R] redacted due to small event counts or to ensure that redacted values could not be derived; RCT, radiotherapy or chemotherapy in past 6 months; SOT, solid organ transplant.

##### Table S6. Incidence rates and hazard ratios for the association between time since vaccination and severe COVID-19.

|  |  |  |  |  |  |  | **Hazard ratio (95% CI)** | | | |
| --- | --- | --- | --- | --- | --- | --- | --- | --- | --- | --- |
| **Wave** | **Subgroup** | **Timing of last COVID-19 vaccine dose** | **N** | **N events** | **Person-years** | **Incidence rate per 1000 person-years (95%CI)** | **Minimally adjusted** | **Extended** | **Fully adjusted** | **Fully adjusted with censoring at next dose** |
| **Delta** | **SOT** | 27+ weeks/unvaccinated | 1,340 | 50 | 258,740 | 70.6 (51-90.1) | 1 (ref) | 1 (ref) | 1 (ref) | 1 (ref) |
|  |  | 13-26 weeks | 990 | 40 | 189,380 | 77.1 (53.2-101.1) | 1.01 (0.66-1.54) | 1.01 (0.66-1.54) | 0.96 (0.63-1.48) | 1.45 (0.9-2.35) |
|  |  | 0-12 weeks | 18,595 | 580 | 3,632,995 | 58.3 (53.6-63.1) | 0.77 (0.57-1.04) | 0.77 (0.57-1.03) | 0.77 (0.57-1.04) | 0.85 (0.62-1.17) |
|  | **BMC** | 27+ weeks/unvaccinated | 6,955 | 105 | 1,344,635 | 28.5 (23.1-34) | 1 (ref) | 1 (ref) | 1 (ref) | 1 (ref) |
|  |  | 13-26 weeks | 6,580 | 135 | 1,249,940 | 39.4 (32.8-46.1) | 0.76 (0.58-0.99) | 0.75 (0.58-0.98) | 0.75 (0.57-0.98) | 0.95 (0.69-1.3) |
|  |  | 0-12 weeks | 100,670 | 1,175 | 19,702,965 | 21.8 (20.5-23) | 0.5 (0.41-0.61) | 0.5 (0.4-0.61) | 0.5 (0.41-0.62) | 0.56 (0.45-0.71) |
|  | **RCT** | 27+ weeks/unvaccinated | 490 | 15 | 88,125 | 62.2 (30.7-93.6) | 1 (ref) | 1 (ref) | 1 (ref) | 1 (ref) |
|  |  | 13-26 weeks | 600 | [R] | [R] | [R] | [R] | [R] | [R] | [R] |
|  |  | 0-12 weeks | 9,735 | 105 | 1,798,465 | 21.3 (17.2-25.4) | 0.38 (0.21-0.69) | 0.37 (0.2-0.67) | 0.4 (0.22-0.73) | 0.4 (0.22-0.75) |
|  | **IMM** | 27+ weeks/unvaccinated | 4,950 | 80 | 972,135 | 30.1 (23.5-36.6) | 1 (ref) | 1 (ref) | 1 (ref) | 1 (ref) |
|  |  | 13-26 weeks | 6,080 | 60 | 1,185,880 | 18.5 (13.8-23.2) | 0.36 (0.26-0.52) | 0.36 (0.26-0.52) | 0.41 (0.29-0.58) | 0.56 (0.38-0.84) |
|  |  | 0-12 weeks | 134,920 | 920 | 26,703,995 | 12.6 (11.8-13.4) | 0.33 (0.26-0.41) | 0.32 (0.26-0.41) | 0.37 (0.29-0.47) | 0.42 (0.32-0.54) |
|  | **IMD** | 27+ weeks/unvaccinated | 18,975 | 215 | 3,710,210 | 21.2 (18.3-24) | 1 (ref) | 1 (ref) | 1 (ref) | 1 (ref) |
|  |  | 13-26 weeks | 9,065 | 120 | 1,751,210 | 25 (20.6-29.5) | 0.53 (0.42-0.67) | 0.53 (0.42-0.67) | 0.47 (0.37-0.6) | 0.61 (0.46-0.8) |
|  |  | 0-12 weeks | 164,620 | 1,110 | 32,421,190 | 12.5 (11.8-13.2) | 0.36 (0.31-0.42) | 0.36 (0.31-0.42) | 0.33 (0.29-0.39) | 0.39 (0.33-0.46) |
| **BA.1/BA.2** | **SOT** | 27+ weeks/unvaccinated | 3,245 | 155 | 419,250 | 135 (113.8-156.3) | 1 (ref) | 1 (ref) | 1 (ref) | 1 (ref) |
|  |  | 13-26 weeks | 960 | 30 | 127,150 | 86.2 (55.3-117) | 0.69 (0.47-1.01) | 0.69 (0.47-1.01) | 0.7 (0.48-1.03) | 0.62 (0.35-1.08) |
|  |  | 0-12 weeks | 15,865 | 635 | 2,086,515 | 111.2 (102.5-119.8) | 0.71 (0.59-0.85) | 0.7 (0.59-0.84) | 0.7 (0.59-0.85) | 0.61 (0.49-0.76) |
|  | **BMC** | 27+ weeks/unvaccinated | 16,405 | 390 | 2,135,745 | 66.7 (60.1-73.3) | 1 (ref) | 1 (ref) | 1 (ref) | 1 (ref) |
|  |  | 13-26 weeks | 3,270 | 50 | 433,495 | 42.1 (30.5-53.8) | 0.73 (0.55-0.97) | 0.73 (0.55-0.97) | 0.75 (0.56-1.01) | 0.87 (0.61-1.24) |
|  |  | 0-12 weeks | 93,030 | 1,705 | 12,307,000 | 50.6 (48.2-53) | 0.5 (0.45-0.56) | 0.5 (0.45-0.56) | 0.53 (0.47-0.59) | 0.45 (0.39-0.52) |
|  | **RCT** | 27+ weeks/unvaccinated | 1,755 | 75 | 213,950 | 128 (99.1-157) | 1 (ref) | 1 (ref) | 1 (ref) | 1 (ref) |
|  |  | 13-26 weeks | 435 | 20 | 55,995 | 130.5 (73.3-187.6) | 0.84 (0.5-1.44) | 0.85 (0.5-1.44) | 0.88 (0.51-1.5) | [R] |
|  |  | 0-12 weeks | 8,815 | 200 | 1,129,790 | 64.7 (55.7-73.6) | 0.51 (0.39-0.68) | 0.51 (0.39-0.67) | 0.51 (0.39-0.68) | 0.44 (0.32-0.61) |
|  | **IMM** | 27+ weeks/unvaccinated | 17,225 | 285 | 2,279,270 | 45.7 (40.4-51) | 1 (ref) | 1 (ref) | 1 (ref) | 1 (ref) |
|  |  | 13-26 weeks | 3,700 | 30 | 494,010 | 22.2 (14.2-30.1) | 0.54 (0.37-0.78) | 0.54 (0.37-0.77) | 0.57 (0.39-0.82) | 0.69 (0.45-1.05) |
|  |  | 0-12 weeks | 120,880 | 1,295 | 16,154,680 | 29.3 (27.7-30.9) | 0.51 (0.45-0.59) | 0.51 (0.45-0.59) | 0.55 (0.48-0.64) | 0.43 (0.36-0.5) |
|  | **IMD** | 27+ weeks/unvaccinated | 39,730 | 535 | 5,251,645 | 37.2 (34.1-40.4) | 1 (ref) | 1 (ref) | 1 (ref) | 1 (ref) |
|  |  | 13-26 weeks | 9,250 | 75 | 1,235,690 | 22.2 (17.2-27.2) | 0.75 (0.59-0.96) | 0.75 (0.59-0.96) | 0.79 (0.62-1.01) | 0.91 (0.68-1.22) |
|  |  | 0-12 weeks | 140,785 | 1,595 | 18,745,140 | 31.1 (29.6-32.6) | 0.47 (0.43-0.53) | 0.48 (0.43-0.53) | 0.48 (0.43-0.53) | 0.41 (0.36-0.46) |
|  |  |  |  |  |  |  |  |  |  |  |
| **JN.1** | **SOT** | 27+ weeks/unvaccinated | 8,655 | 50 | 1,005,545 | 18.2 (13.1-23.2) | 1 (ref) | 1 (ref) | 1 (ref) | 1 (ref) |
|  |  | 13-26 weeks | 440 | [R] | [R] | [R] | [R] | [R] | [R] | [R] |
|  |  | 0-12 weeks | 12,255 | 85 | 1,428,730 | 21.7 (17.1-26.3) | 0.96 (0.66-1.4) | 0.91 (0.62-1.33) | 0.88 (0.6-1.3) | 0.88 (0.59-1.3) |
|  | **BMC** | 27+ weeks/unvaccinated | 41,890 | 200 | 4,859,510 | 15 (12.9-17.1) | 1 (ref) | 1 (ref) | 1 (ref) | 1 (ref) |
|  |  | 13-26 weeks | 2,010 | 15 | 230,920 | 23.7 (11.7-35.7) | 0.91 (0.52-1.59) | 0.89 (0.51-1.57) | 0.89 (0.51-1.57) | [R] |
|  |  | 0-12 weeks | 76,400 | 390 | 8,889,480 | 16 (14.4-17.6) | 0.64 (0.54-0.77) | 0.62 (0.52-0.75) | 0.64 (0.53-0.77) | 0.64 (0.53-0.77) |
|  | **RCT** | 27+ weeks/unvaccinated | 5,020 | 55 | 562,130 | 35.7 (26.3-45.2) | 1 (ref) | 1 (ref) | 1 (ref) | 1 (ref) |
|  |  | 13-26 weeks | 190 | [R] | [R] | [R] | [R] | [R] | [R] | [R] |
|  |  | 0-12 weeks | 7,400 | 55 | 837,755 | 24 (17.6-30.3) | 0.6 (0.4-0.9) | 0.62 (0.41-0.92) | 0.68 (0.44-1.03) | 0.68 (0.44-1.03) |
|  | **IMM** | 27+ weeks/unvaccinated | 51,265 | 90 | 6,011,730 | 5.5 (4.3-6.6) | 1 (ref) | 1 (ref) | 1 (ref) | 1 (ref) |
|  |  | 13-26 weeks | 2,735 | [R] | [R] | [R] | [R] | [R] | [R] | [R] |
|  |  | 0-12 weeks | 94,815 | 210 | 11,120,780 | 6.9 (6-7.8) | 0.68 (0.53-0.89) | 0.68 (0.52-0.88) | 0.68 (0.52-0.88) | 0.69 (0.52-0.9) |
|  | **IMD** | 27+ weeks/unvaccinated | 99,255 | 180 | 11,598,260 | 5.7 (4.8-6.5) | 1 (ref) | 1 (ref) | 1 (ref) | 1 (ref) |
|  |  | 13-26 weeks | 2,750 | [R] | [R] | [R] | [R] | [R] | [R] | [R] |
|  |  | 0-12 weeks | 103,460 | 305 | 12,064,635 | 9.2 (8.2-10.3) | 0.72 (0.59-0.88) | 0.71 (0.58-0.87) | 0.68 (0.55-0.82) | 0.68 (0.55-0.83) |

Minimally adjusted models adjusted for age (using a restricted cubic spline with four knots) and sex. Extended models further adjusted for prior infection. Fully adjusted models further adjusting for ethnicity, index of multiple deprivation quintile, and comorbidity count. All models included region as a stratification variable. BMC, bone marrow compromising condition; IMD, primary or acquired immunodeficiency; IMM, immunosuppressive medication in past 6 months; [R], redacted due to small event counts; RCT, radiotherapy or chemotherapy in past 6 months; SOT, solid organ transplant.

##### Table S7. Incidence rates and hazard ratios for the association between time since vaccination and COVID-19-related mortality.

|  |  |  |  |  |  | **Incidence rate per 1000 person-years (95%CI)** | **Hazard ratio (95% CI)** | | |
| --- | --- | --- | --- | --- | --- | --- | --- | --- | --- |
| **Wave** | **Subgroup** | **Timing of last COVID-19 vaccine dose** | **N** | **N events** | **Person-years** |  | **Minimally adjusted** | **Extended** | **Fully adjusted** |
| **Delta** | **SOT** | 27+ weeks/unvaccinated | 1,340 | [R] | [R] | [R] | [R] | [R] | [R] |
|  |  | 13-26 weeks | 990 | [R] | [R] | [R] | [R] | [R] | [R] |
|  |  | 0-12 weeks | 18,595 | [R] | [R] | [R] | [R] | [R] | [R] |
|  | **BMC** | 27+ weeks/unvaccinated | 6,955 | 30 | 1,351,595 | 8.1 (5.2-11) | 1 (ref) | 1 (ref) | 1 (ref) |
|  |  | 13-26 weeks | 6,580 | 40 | 1,257,445 | 11.6 (8-15.2) | 0.49 (0.3-0.81) | 0.49 (0.3-0.81) | 0.48 (0.29-0.79) |
|  |  | 0-12 weeks | 100,670 | 310 | 19,768,475 | 5.7 (5.1-6.4) | 0.35 (0.24-0.52) | 0.35 (0.24-0.52) | 0.35 (0.23-0.52) |
|  | **RCT** | 27+ weeks/unvaccinated | 490 | [R] | [R] | [R] | [R] | [R] | [R] |
|  |  | 13-26 weeks | 600 | [R] | [R] | [R] | [R] | [R] | [R] |
|  |  | 0-12 weeks | 9,735 | [R] | [R] | [R] | [R] | [R] | [R] |
|  | **IMM** | 27+ weeks/unvaccinated | 4,950 | 20 | 977,970 | 7.5 (4.2-10.7) | 1 (ref) | 1 (ref) | 1 (ref) |
|  |  | 13-26 weeks | 6,080 | 15 | 1,189,815 | 4.6 (2.3-6.9) | 0.17 (0.08-0.35) | 0.17 (0.08-0.35) | 0.22 (0.11-0.45) |
|  |  | 0-12 weeks | 134,920 | 190 | 26,759,490 | 2.6 (2.2-3) | 0.18 (0.11-0.29) | 0.18 (0.11-0.29) | 0.23 (0.14-0.38) |
|  | **IMD** | 27+ weeks/unvaccinated | 18,975 | 40 | 3,724,655 | 3.9 (2.7-5.1) | 1 (ref) | 1 (ref) | 1 (ref) |
|  |  | 13-26 weeks | 9,065 | 30 | 1,758,260 | 6.2 (4-8.5) | 0.41 (0.25-0.66) | 0.4 (0.25-0.66) | 0.36 (0.22-0.58) |
|  |  | 0-12 weeks | 164,620 | 265 | 32,485,430 | 3 (2.6-3.3) | 0.31 (0.22-0.44) | 0.31 (0.22-0.44) | 0.29 (0.2-0.41) |
| **BA.1/BA.2** | **SOT** | 27+ weeks/unvaccinated | 3,245 | [R] | [R] | [R] | [R] | [R] | [R] |
|  |  | 13-26 weeks | 960 | [R] | [R] | [R] | [R] | [R] | [R] |
|  |  | 0-12 weeks | 15,865 | [R] | [R] | [R] | [R] | [R] | [R] |
|  | **BMC** | 27+ weeks/unvaccinated | 16,405 | 60 | 2,159,690 | 10.1 (7.6-12.7) | 1 (ref) | 1 (ref) | 1 (ref) |
|  |  | 13-26 weeks | 3,270 | [R] | [R] | [R] | [R] | [R] | [R] |
|  |  | 0-12 weeks | 93,030 | 290 | 12,394,355 | 8.5 (7.6-9.5) | 0.41 (0.31-0.54) | 0.41 (0.31-0.54) | 0.43 (0.32-0.58) |
|  | **RCT** | 27+ weeks/unvaccinated | 1,755 | [R] | [R] | [R] | [R] | [R] | [R] |
|  |  | 13-26 weeks | 435 | [R] | [R] | [R] | [R] | [R] | [R] |
|  |  | 0-12 weeks | 8,815 | [R] | [R] | [R] | [R] | [R] | [R] |
|  | **IMM** | 27+ weeks/unvaccinated | 17,225 | 45 | 2,297,480 | 7.2 (5.1-9.2) | 1 (ref) | 1 (ref) | 1 (ref) |
|  |  | 13-26 weeks | 3,700 | [R] | [R] | [R] | [R] | [R] | [R] |
|  |  | 0-12 weeks | 120,880 | 100 | 16,227,340 | 2.3 (1.8-2.7) | 0.17 (0.12-0.24) | 0.17 (0.12-0.24) | 0.2 (0.14-0.29) |
|  | **IMD** | 27+ weeks/unvaccinated | 39,730 | 75 | 5,285,175 | 5.2 (4-6.4) | 1 (ref) | 1 (ref) | 1 (ref) |
|  |  | 13-26 weeks | 9,250 | [R] | [R] | [R] | [R] | [R] | [R] |
|  |  | 0-12 weeks | 140,785 | 185 | 18,830,055 | 3.6 (3.1-4.1) | 0.28 (0.21-0.37) | 0.28 (0.21-0.37) | 0.3 (0.22-0.39) |

There were insufficient event counts in the JN.1 wave to reliably estimate hazard ratios for COVID-19-related mortality in any subgroup. Minimally adjusted models adjusted for age (using a restricted cubic spline with four knots) and sex. Extended models further adjusted for prior infection. Fully adjusted models further adjusting for ethnicity, index of multiple deprivation quintile, and comorbidity count. All models included region as a stratification variable. BMC, bone marrow compromising condition; IMD, primary or acquired immunodeficiency; IMM, immunosuppressive medication in past 6 months; [R], redacted due to small event counts or to ensure that redacted values could not be derived; RCT, radiotherapy or chemotherapy in past 6 months; SOT, solid organ transplant.

**
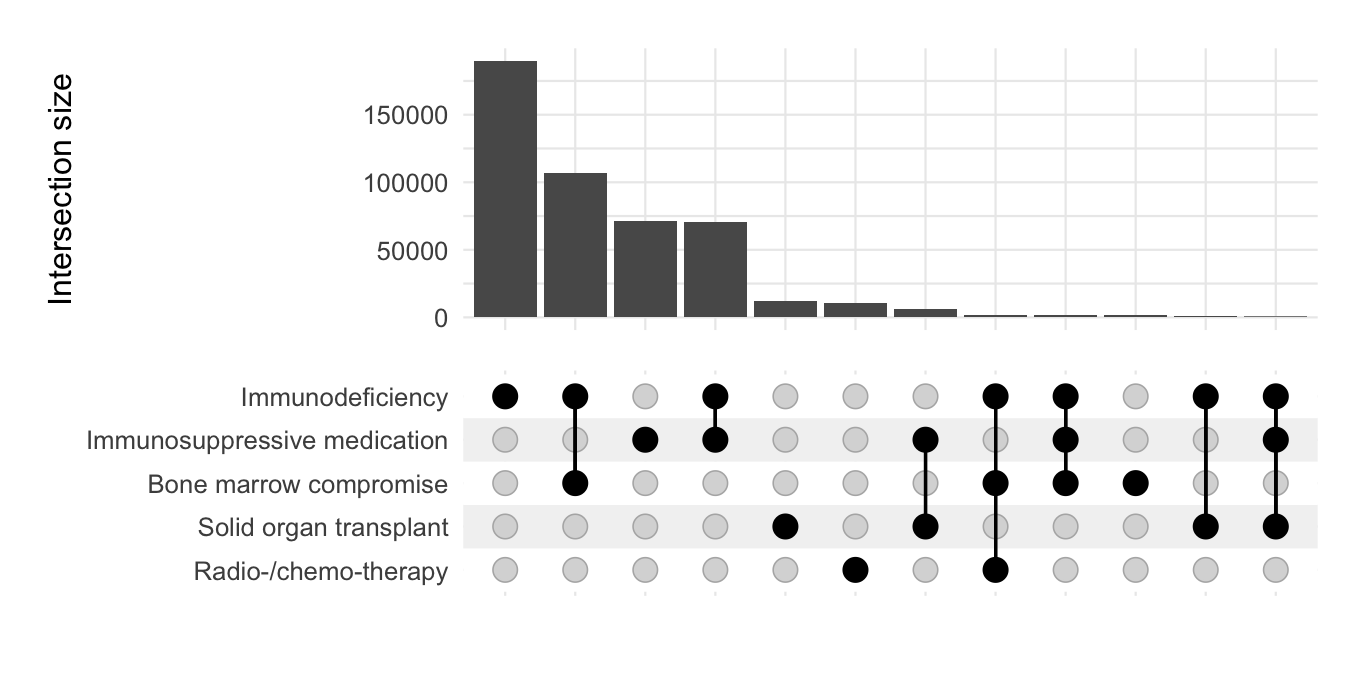
**

**Fig S1. Intersection of immunocompromised subgroups prior to hierarchical subgroup assignment.** Data shown for the start of the Omicron BA.1/BA.2 wave (15 December 2021). Intersections with a minimum size of 500 are shown. See **Table S3** for underlying data.


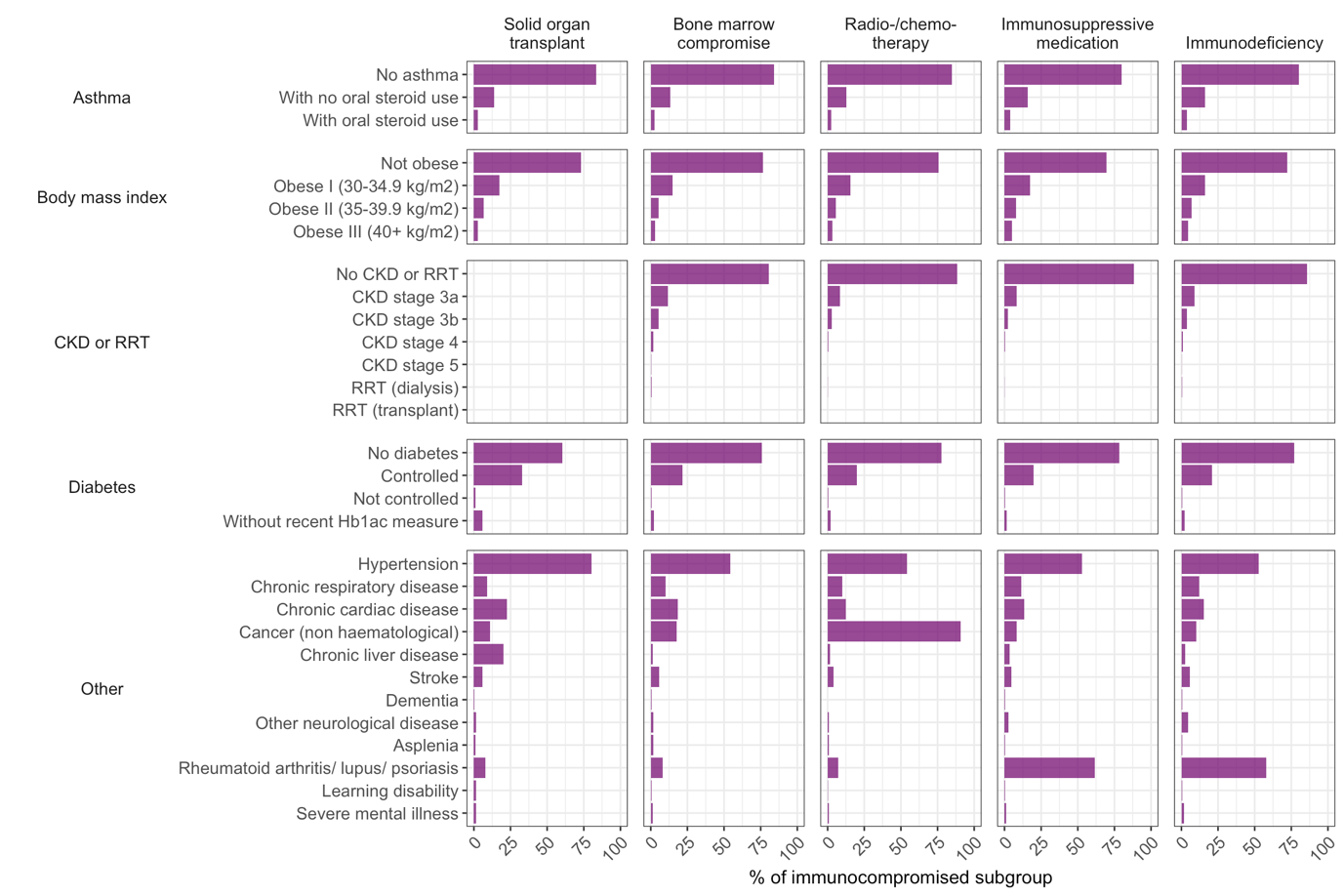


*

**Fig S2. Comorbidity prevalence by immunocompromised subgroup.** Data shown for the start of the JN.1 wave (04 December 2023). See **File S1** for underlying data. * CKD or RRT status excluded as a covariate for individuals with solid organ transplant due to the high prevalence of kidney transplant recipients in this subgroup. CKD, chronic kidney disease; RRT, renal replacement therapy.

**
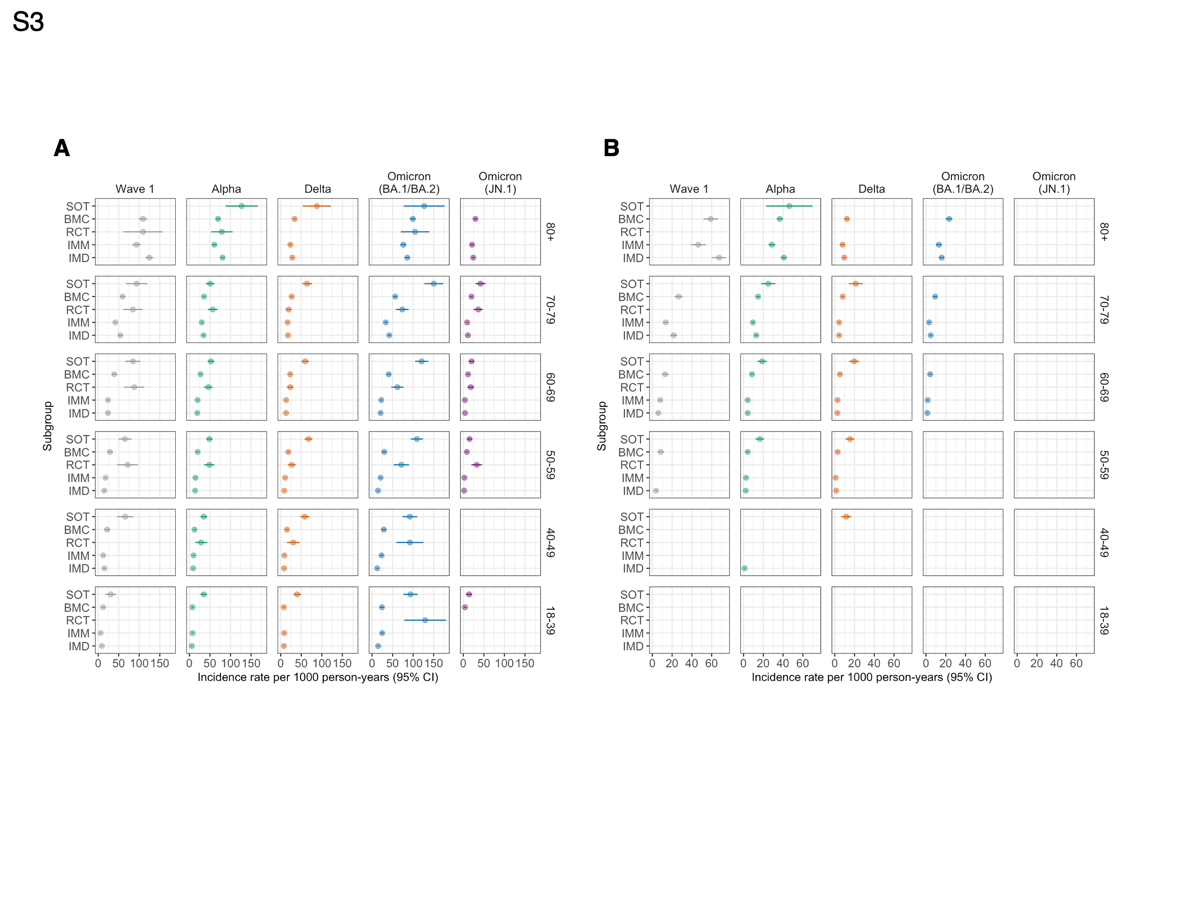
**

**Fig S3. Age-stratified absolute incidence of severe COVID-19 by immunocompromised subgroup.** Absolute rates of **(A)** severe COVID-19 and **(B)** COVID-19-related mortality by immunocompromised subgroup over time. Unplotted values indicate that estimates were redacted due to small counts or to ensure that redacted values could not be derived. BMC, bone marrow compromising condition; CI, confidence interval; IMD, primary or acquired immunodeficiency; IMM, immunosuppressive medication in past 6 months; RCT, radiotherapy or chemotherapy in past 6 months; SOT, solid organ transplant.

**Fig S4. Association between number of prior doses and severe COVID-19. (A)** Baseline vaccination status at the start of each wave after COVID-19 vaccine implementation. See **File S1** for underlying data. **(B)** Relative hazard of severe COVID-19 based on models adjusting for age, sex, prior infection, ethnicity, deprivation quintile, and comorbidity count, with region as a stratification factor. Unplotted values indicate that estimates were redacted due to small counts or to ensure that redacted values could not be derived. See **File S2** for underlying data. CI, confidence interval.

**Fig S5. Association between recorded prior infection status and severe COVID-19. (A)** Prior infection status based on primary and secondary care records at the start of each wave (excluding wave 1). See **File S1** for underlying data. **(B)** Relative hazard of severe COVID-19 based on models adjusting for age, sex, time since last vaccine dose, ethnicity, deprivation quintile, and comorbidity count, with region as a stratification factor. Unplotted values indicate that estimates were redacted due to small counts or to ensure that redacted values could not be derived. See **File S2** for underlying data. CI, confidence interval.

**
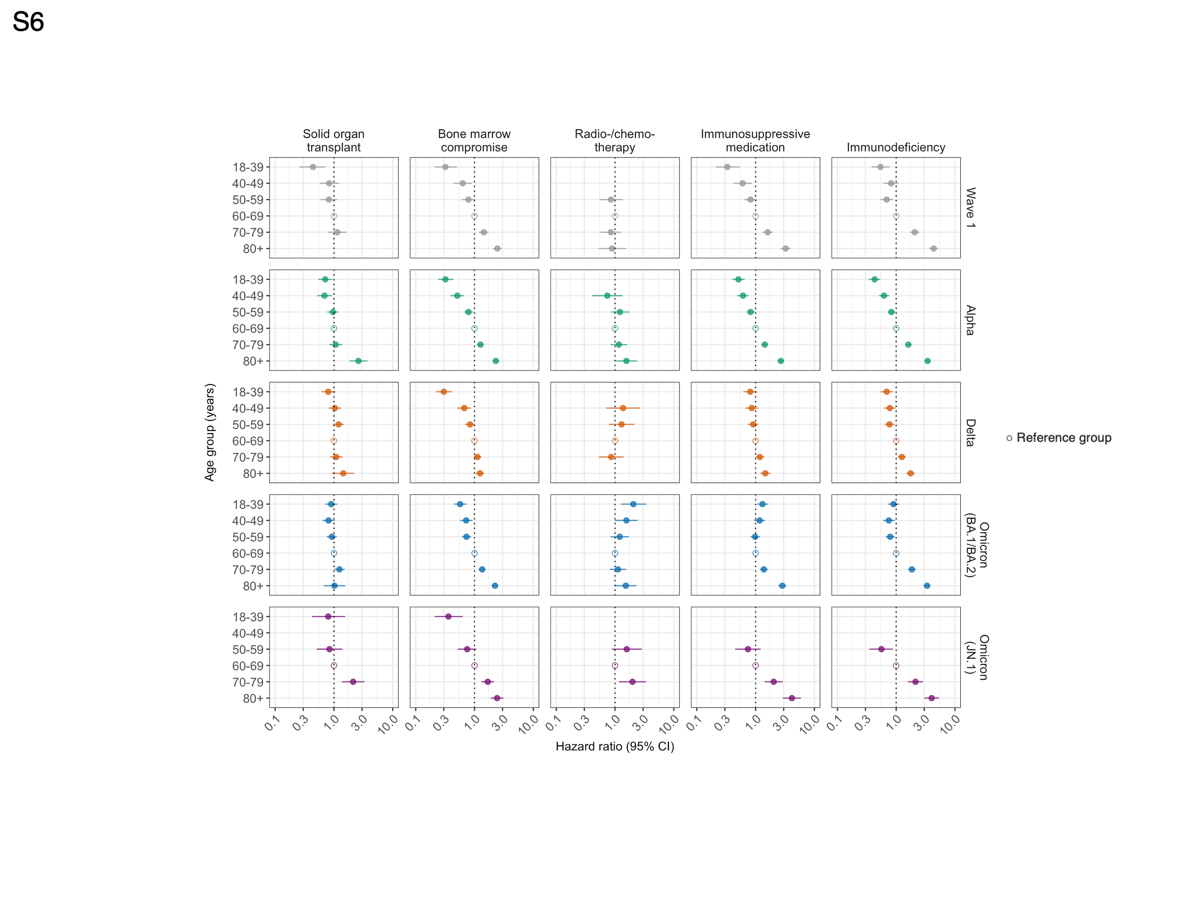
**

**Fig S6. Association between age and severe COVID-19 over time.** Relative hazard of severe COVID-19 based on models adjusting for sex, time since last vaccine dose, prior infection, ethnicity, deprivation quintile, and comorbidity count, with region as a stratification factor. Unplotted values indicate that estimates were redacted due to small counts or to ensure that redacted values could not be derived. See **File S2** for underlying data. CI, confidence interval.

**
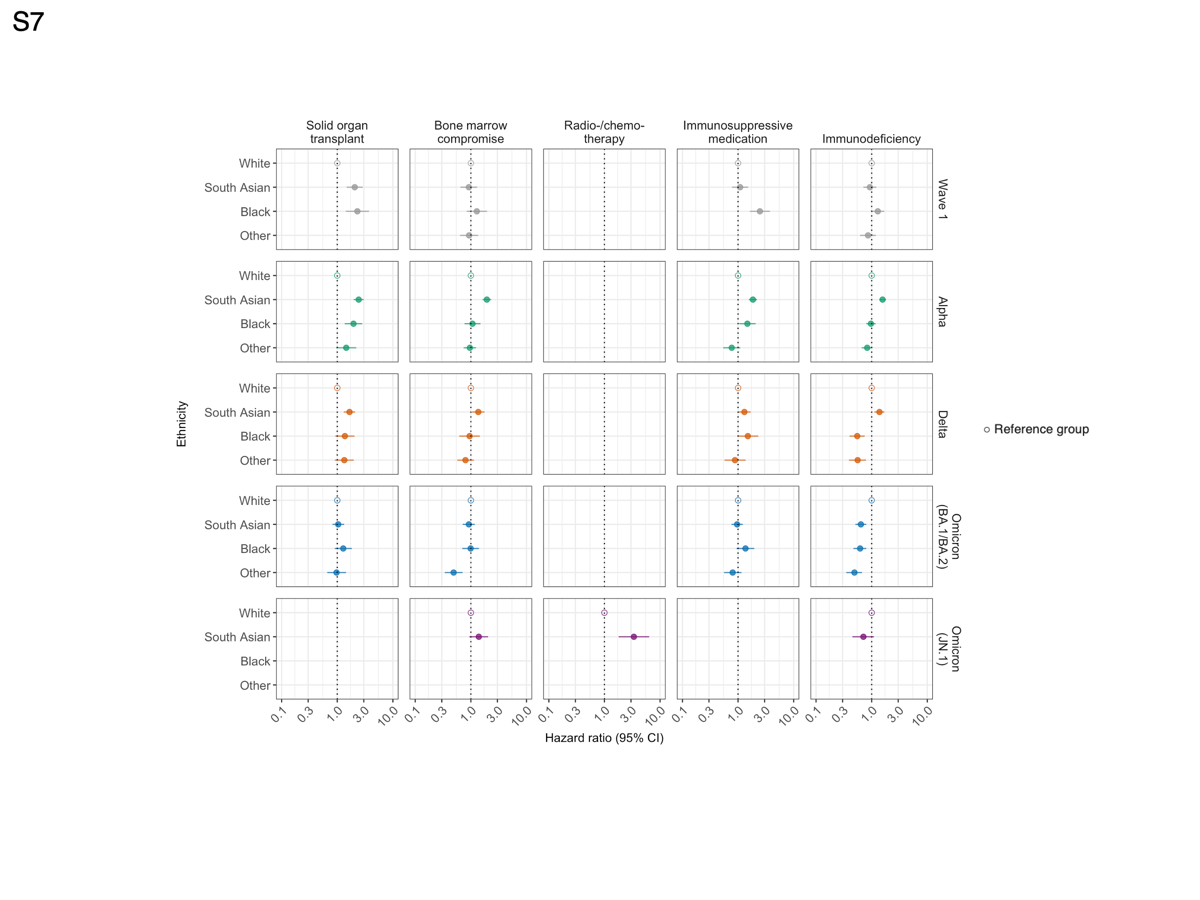
**

**S7 Fig. Association between ethnicity and severe COVID-19 over time.** Relative hazard of severe COVID-19 based on models adjusting for age, sex, time since last vaccine dose, prior infection, deprivation quintile, and comorbidity count, with region as a stratification factor. Unplotted values indicate that estimates were redacted due to small counts or to ensure that redacted values could not be derived. See **File S2** for underlying data. CI, confidence interval.

**
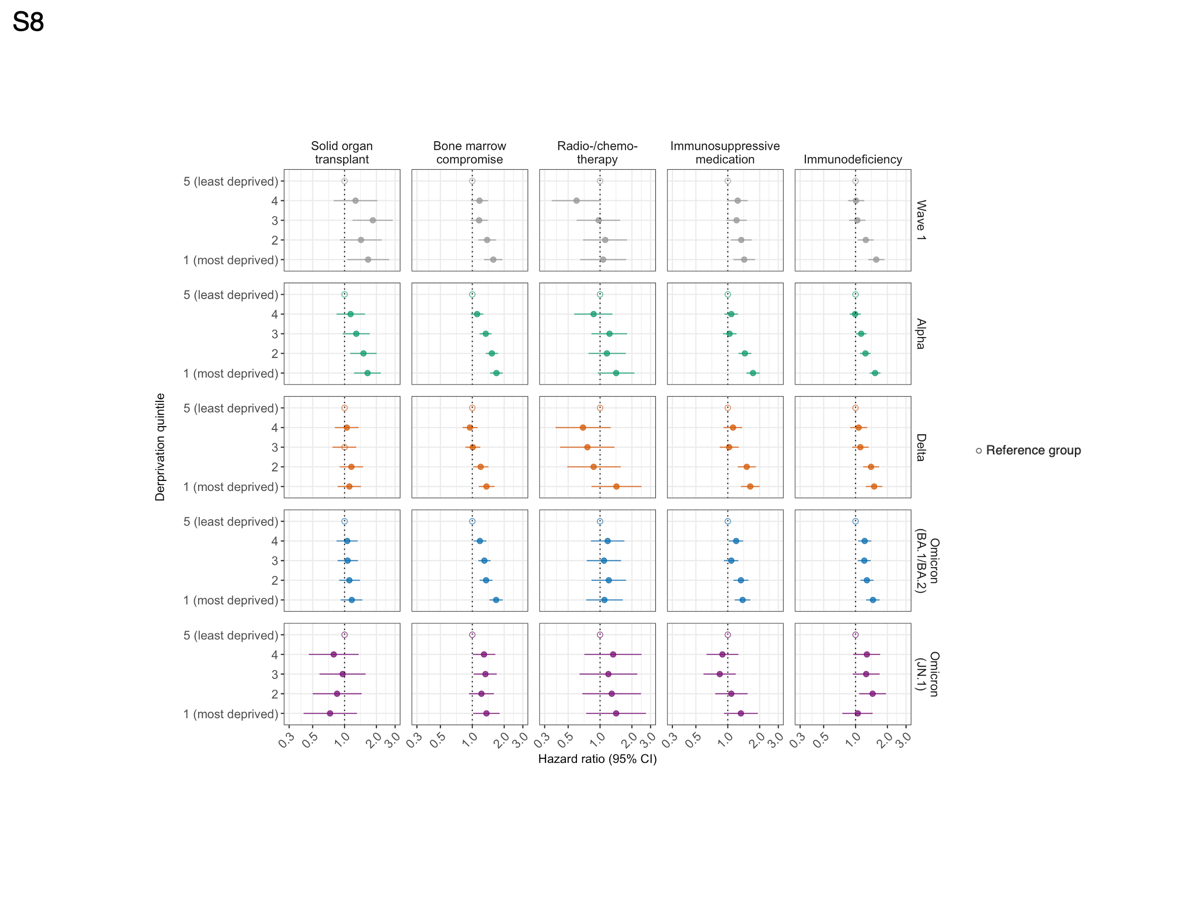
**

**S8 Fig. Association between index of multiple deprivation quintile and severe COVID-19 over time.** Relative hazard of severe COVID-19 based on models adjusting for age, sex, time since last vaccine dose, prior infection, ethnicity, and comorbidity count, with region as a stratification factor.

See **File S2** for underlying data. CI, confidence interval.

**
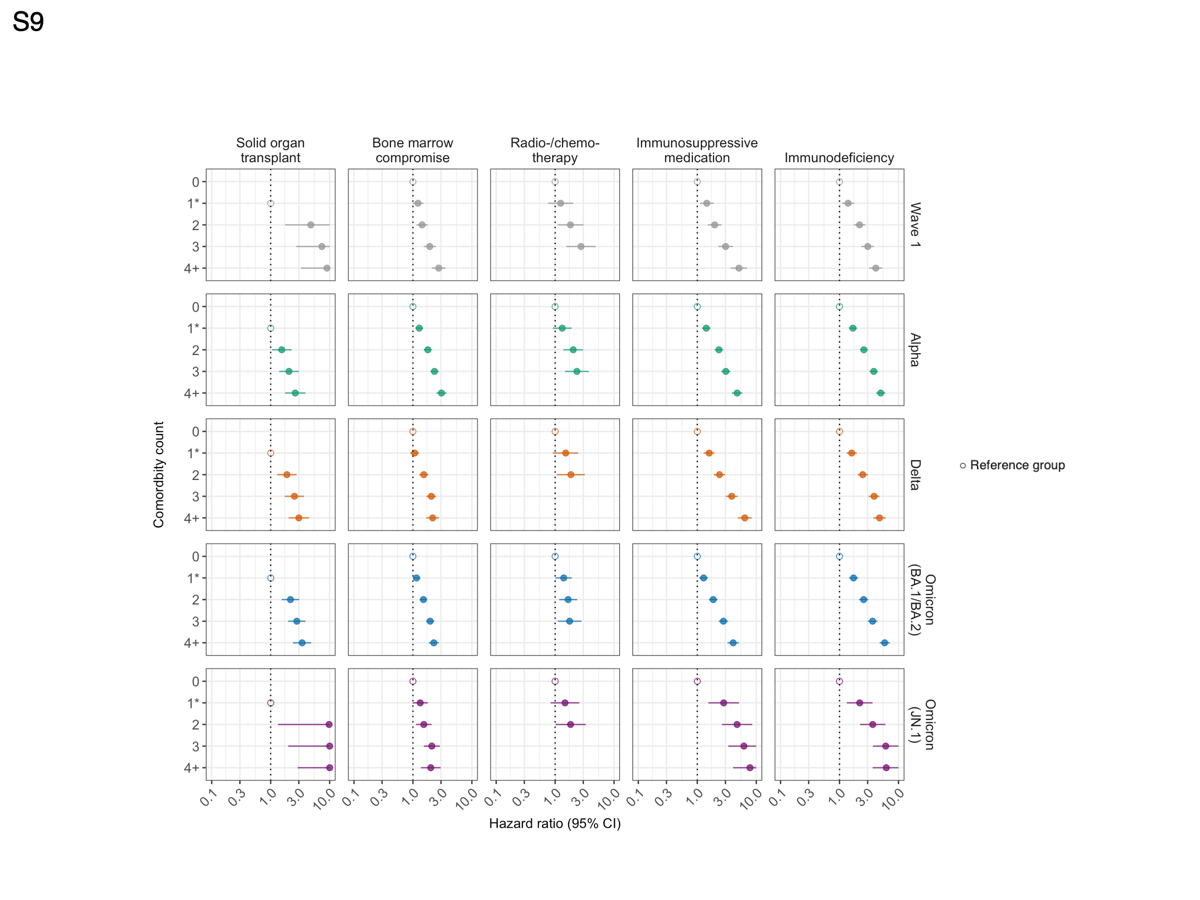
**

**S9 Fig. Association between comorbidity count and severe COVID-19 over time.** Relative hazard of severe COVID-19 based on models adjusting for age, sex, time since last vaccine dose, prior infection, ethnicity, and deprivation quintile, with region as a stratification factor. Unplotted values indicate that estimates were redacted due to small counts or to ensure that redacted values could not be derived. See **File S2** for underlying data. CI, confidence interval. * ≤1 comorbidity used as baseline group for individuals with solid organ transplants due to the high prevalence of comorbidities in this subgroup.

**
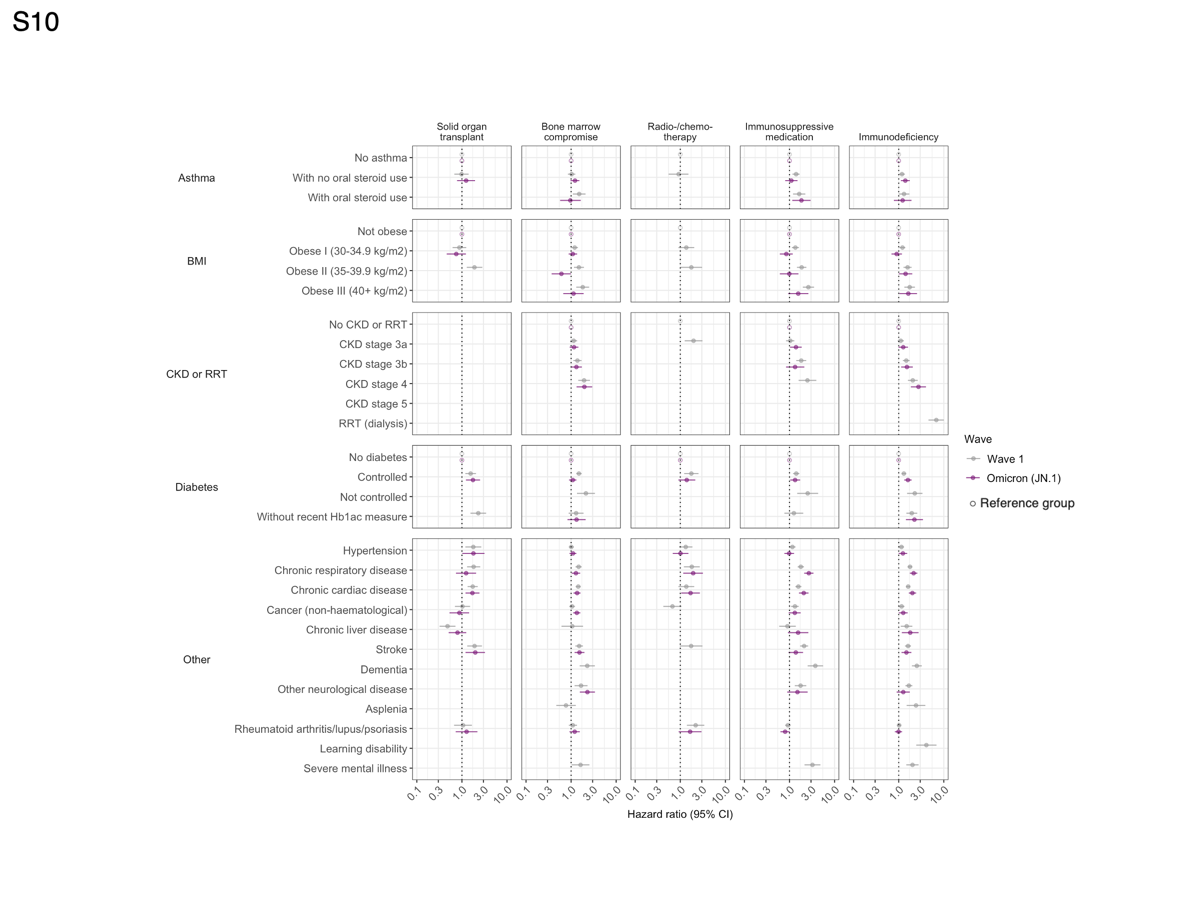
**

*

**Fig S10. Association between individual clinical risk groups and severe COVID-19 in wave 1 and the JN.1 wave.** Relative hazard of severe COVID-19 based on models adjusting for age, sex, time since last vaccine dose, prior infection, ethnicity, and deprivation quintile, and (for the subset of variables that did not contribute to its calculation) comorbidity count, with region as a stratification factor. Unplotted values indicate that estimates were redacted due to small counts or to ensure that redacted values could not be derived. See **File S2** for underlying data. BMI, body mass index; CI, confidence interval; CKD, chronic kidney disease; RRT, renal replacement therapy. * CKD or RRT status excluded as a covariate for individuals with solid organ transplant due to the high prevalence of kidney transplant recipients in this subgroup.

**
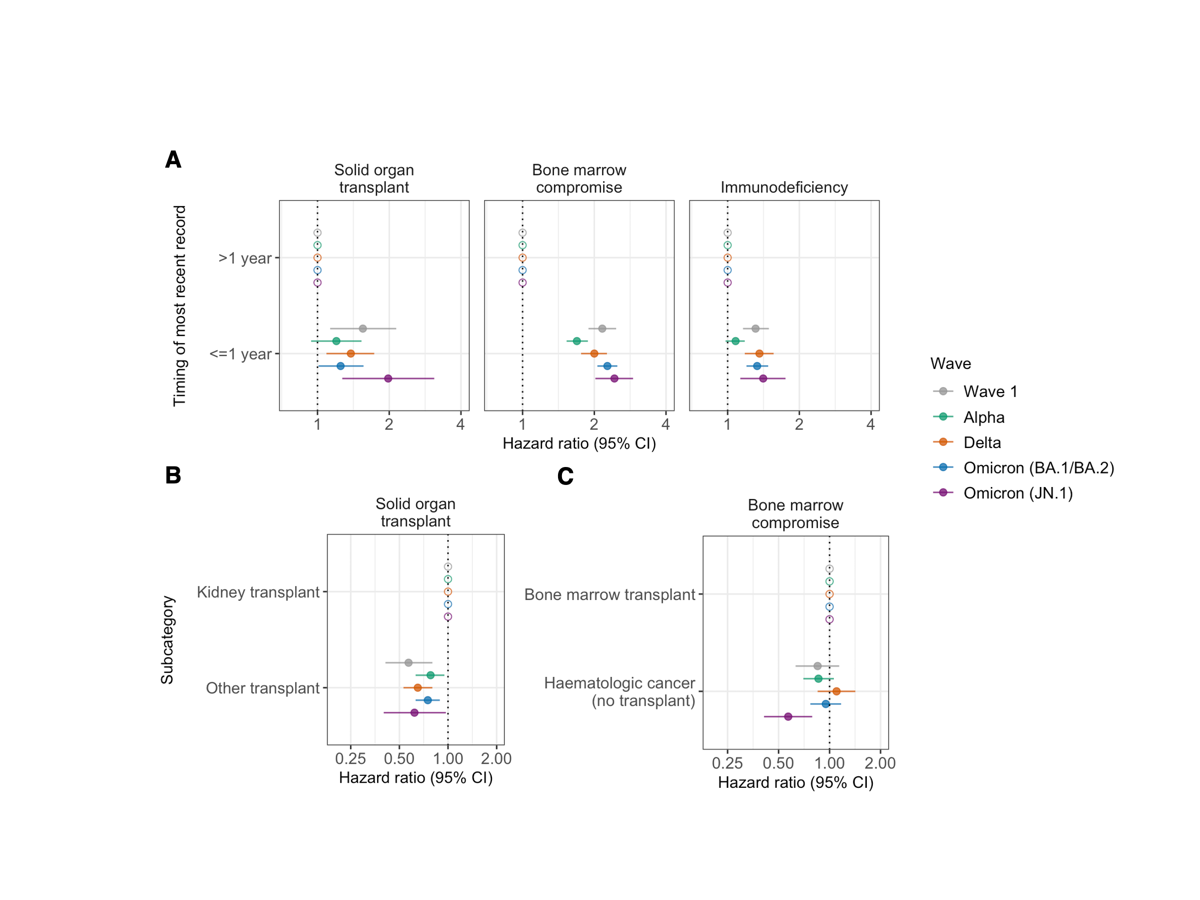
**

**S11 Fig. Association between immunocompromised** **subcategory and severe COVID-19.** Relative hazard of severe COVID-19 based on models adjusting for age, sex, time since last vaccine dose, prior infection, ethnicity, and deprivation quintile, and comorbidity count, with region as a stratification factor. Data are shown for **(A)** timing of most recent primary care record, **(B)** solid organ transplant subcategory, and **(C)** bone marrow compromise subcategory. See **File S2** for underlying data. BMI, body mass index; CI, confidence interval; CKD, chronic kidney disease; RRT, renal replacement therapy.

**The RECORD statement – checklist of items, extended from the STROBE statement, that should be reported in observational studies using routinely collected health data.**

|  | **Item No.** | **STROBE items** | **Location in manuscript where items are reported** | **RECORD items** | **Location in manuscript where items are reported** |
| --- | --- | --- | --- | --- | --- |
| **Title and abstract** | | | | | |
|  | 1 | (a) Indicate the study’s design with a commonly used term in the title or the abstract (b) Provide in the abstract an informative and balanced summary of what was done and what was found | Title, Abstract | RECORD 1.1: The type of data used should be specified in the title or abstract. When possible, the name of the databases used should be included.  RECORD 1.2: If applicable, the geographic region and timeframe within which the study took place should be reported in the title or abstract.  RECORD 1.3: If linkage between databases was conducted for the study, this should be clearly stated in the title or abstract. | Title, Abstract  Title  N/A (single database with established linkages) |
| **Introduction** | | | | | |
| Background rationale | 2 | Explain the scientific background and rationale for the investigation being reported | Background |  |  |
| Objectives | 3 | State specific objectives, including any prespecified hypotheses | Background |  |  |
| **Methods** | | | | | |
| Study Design | 4 | Present key elements of study design early in the paper | Study population subheading |  |  |
| Setting | 5 | Describe the setting, locations, and relevant dates, including periods of recruitment, exposure, follow-up, and data collection | Study population, Outcomes, Covariates, Table S1 |  |  |
| Participants | 6 | *(a) Cohort study* - Give the eligibility criteria, and the sources and methods of selection of participants. Describe methods of follow-up  *Case-control study* - Give the eligibility criteria, and the sources and methods of case ascertainment and control selection. Give the rationale for the choice of cases and controls  *Cross-sectional study* - Give the eligibility criteria, and the sources and methods of selection of participants  *(b) Cohort study* - For matched studies, give matching criteria and number of exposed and unexposed  *Case-control study* - For matched studies, give matching criteria and the number of controls per case | Data sources, Study population, Statistical analysis  N/A | RECORD 6.1: The methods of study population selection (such as codes or algorithms used to identify subjects) should be listed in detail. If this is not possible, an explanation should be provided.  RECORD 6.2: Any validation studies of the codes or algorithms used to select the population should be referenced. If validation was conducted for this study and not published elsewhere, detailed methods and results should be provided.  RECORD 6.3: If the study involved linkage of databases, consider use of a flow diagram or other graphical display to demonstrate the data linkage process, including the number of individuals with linked data at each stage. | Study population, Github repository  Table S1 provides OpenCodelists IDs for all codelists used in study; the methodology underpinning each codelist is described in full on OpenCodelists.  N/A (study uses established linkages in OpenSAFELY) |
| Variables | 7 | Clearly define all outcomes, exposures, predictors, potential confounders, and effect modifiers. Give diagnostic criteria, if applicable. | Outcomes, Covariates | RECORD 7.1: A complete list of codes and algorithms used to classify exposures, outcomes, confounders, and effect modifiers should be provided. If these cannot be reported, an explanation should be provided. | Table S1, Github repository |
| Data sources/ measurement | 8 | For each variable of interest, give sources of data and details of methods of assessment (measurement).  Describe comparability of assessment methods if there is more than one group | Table S1  N/A |  |  |
| Bias | 9 | Describe any efforts to address potential sources of bias | Covariates (description of handling of missing data), Statistical analysis (sequential modelling approach and sensitivity analysis) |  |  |
| Study size | 10 | Explain how the study size was arrived at | Table S2 |  |  |
| Quantitative variables | 11 | Explain how quantitative variables were handled in the analyses. If applicable, describe which groupings were chosen, and why | Table S1 |  |  |
| Statistical methods | 12 | (a) Describe all statistical methods, including those used to control for confounding  (b) Describe any methods used to examine subgroups and interactions  (c) Explain how missing data were addressed  (d) *Cohort study* - If applicable, explain how loss to follow-up was addressed  *Case-control study* - If applicable, explain how matching of cases and controls was addressed  *Cross-sectional study* - If applicable, describe analytical methods taking account of sampling strategy  (e) Describe any sensitivity analyses | Study population (b), Covariates (c), Statistical analysis (a, b, d, e) |  |  |
| Data access and cleaning methods |  | .. |  | RECORD 12.1: Authors should describe the extent to which the investigators had access to the database population used to create the study population.  RECORD 12.2: Authors should provide information on the data cleaning methods used in the study. | Information governance, Data sharing  Table S1 |
| Linkage |  | .. |  | RECORD 12.3: State whether the study included person-level, institutional-level, or other data linkage across two or more databases. The methods of linkage and methods of linkage quality evaluation should be provided. | Data sources |
| **Results** | | | | | |
| Participants | 13 | (a) Report the numbers of individuals at each stage of the study (*e.g.*, numbers potentially eligible, examined for eligibility, confirmed eligible, included in the study, completing follow-up, and analysed)  (b) Give reasons for non-participation at each stage.  (c) Consider use of a flow diagram | Study population, Table S2 | RECORD 13.1: Describe in detail the selection of the persons included in the study (*i.e.,* study population selection) including filtering based on data quality, data availability and linkage. The selection of included persons can be described in the text and/or by means of the study flow diagram. | Table S2 |
| Descriptive data | 14 | (a) Give characteristics of study participants (*e.g.*, demographic, clinical, social) and information on exposures and potential confounders  (b) Indicate the number of participants with missing data for each variable of interest  (c) *Cohort study* - summarise follow-up time (*e.g.*, average and total amount) | Figure 2 (a), Table S4 (a, b), File S1 (a, b), File S2 (c), Tables S5–S7 (c) |  |  |
| Outcome data | 15 | *Cohort study* - Report numbers of outcome events or summary measures over time  *Case-control study* - Report numbers in each exposure category, or summary measures of exposure  *Cross-sectional study* - Report numbers of outcome events or summary measures | File S2, Tables S5–S7 |  |  |
| Main results | 16 | (a) Give unadjusted estimates and, if applicable, confounder-adjusted estimates and their precision (e.g., 95% confidence interval). Make clear which confounders were adjusted for and why they were included  (b) Report category boundaries when continuous variables were categorized  (c) If relevant, consider translating estimates of relative risk into absolute risk for a meaningful time period | File S2 (a, b, c), Tables S5–S7 (a, b, c), absolute and relative risks are discussed under various Results subheadings |  |  |
| Other analyses | 17 | Report other analyses done—e.g., analyses of subgroups and interactions, and sensitivity analyses | File S2 (sensitivity analyses), Association between recent vaccination and COVID-19 |  |  |
| **Discussion** | | | | | |
| Key results | 18 | Summarise key results with reference to study objectives | Discussion (first paragraph) |  |  |
| Limitations | 19 | Discuss limitations of the study, taking into account sources of potential bias or imprecision. Discuss both direction and magnitude of any potential bias | Discussion (limitations paragraph) | RECORD 19.1: Discuss the implications of using data that were not created or collected to answer the specific research question(s). Include discussion of misclassification bias, unmeasured confounding, missing data, and changing eligibility over time, as they pertain to the study being reported. | Discussion (limitations paragraph) |
| Interpretation | 20 | Give a cautious overall interpretation of results considering objectives, limitations, multiplicity of analyses, results from similar studies, and other relevant evidence | Discussion |  |  |
| Generalisability | 21 | Discuss the generalisability (external validity) of the study results | Discussion (limitations paragraph) |  |  |
| **Other Information** | | | | | |
| Funding | 22 | Give the source of funding and the role of the funders for the present study and, if applicable, for the original study on which the present article is based | Funding |  |  |
| Accessibility of protocol, raw data, and programming code |  | .. |  | RECORD 22.1: Authors should provide information on how to access any supplemental information such as the study protocol, raw data, or programming code. | Link provided for Github repository containing study protocol, codelists, and code |

*Reference: Benchimol EI, Smeeth L, Guttmann A, Harron K, Moher D, Petersen I, Sørensen HT, von Elm E, Langan SM, the RECORD Working Committee. The REporting of studies Conducted using Observational Routinely-collected health Data (RECORD) Statement. *PLoS Medicine* 2015; in press.

*Checklist is protected under Creative Commons Attribution ([CC BY](http://creativecommons.org/licenses/by/4.0/)) license.
